# Advances in Respiratory Monitoring: A Comprehensive Review of Wearable and Remote Technologies

**DOI:** 10.3390/bios14020090

**Published:** 2024-02-06

**Authors:** Diana Vitazkova, Erik Foltan, Helena Kosnacova, Michal Micjan, Martin Donoval, Anton Kuzma, Martin Kopani, Erik Vavrinsky

**Affiliations:** 1 Institute of Electronics and Photonics, Faculty of Electrical Engineering and Information Technology, Slovak University of Technology, Ilkovicova 3, 81219 Bratislava, Slovakia; erik.foltan@stuba.sk (E.F.); helena.kosnacova@stuba.sk (H.K.); michal.micjan@stuba.sk (M.M.); martin.donoval@stuba.sk (M.D.); anton.kuzma@stuba.sk (A.K.); 2 Department of Simulation and Virtual Medical Education, Faculty of Medicine, Comenius University, Sasinkova 4, 81272 Bratislava, Slovakia; 3 Institute of Medical Physics, Biophysics, Informatics and Telemedicine, Faculty of Medicine, Comenius University, Sasinkova 2, 81272 Bratislava, Slovakia; martin.kopani@fmed.uniba.sk

**Keywords:** wearable devices, respiration monitoring, thorax movement, impedance, airflow, acoustic methods

## Abstract

This article explores the importance of wearable and remote technologies in healthcare. The focus highlights its potential in continuous monitoring, examines the specificity of the issue, and offers a view of proactive healthcare. Our research describes a wide range of device types and scientific methodologies, starting from traditional chest belts to their modern alternatives and cutting-edge bioamplifiers that distinguish breathing from chest impedance variations. We also investigated innovative technologies such as the monitoring of thorax micromovements based on the principles of seismocardiography, ballistocardiography, remote camera recordings, deployment of integrated optical fibers, or extraction of respiration from cardiovascular variables. Our review is extended to include acoustic methods and breath and blood gas analysis, providing a comprehensive overview of different approaches to respiratory monitoring. The topic of monitoring respiration with wearable and remote electronics is currently the center of attention of researchers, which is also reflected by the growing number of publications. In our manuscript, we offer an overview of the most interesting ones.

## 1. Introduction

Respiratory diseases are significant contributors to mortality and disability within populations [1], which was especially underlined by the global impact of the COVID-19 pandemic. In developed nations, asthma holds the position as the most prevalent respiratory disease, closely followed by chronic obstructive pulmonary disease (COPD), both significantly compromising the quality of life and frequently resulting in premature death. The World Health Organization reports that 262 million people worldwide had asthma in 2019 [2]. In that year, asthma globally caused approximately 460,000 deaths annually. The trend from 1990 to 2019 has shown no significant variation in the global mortality rate due to this disease. Throughout Europe, the prevalence of asthma in adults varies, ranging from 5.1% to 8.2%. The data on deaths caused by asthma in Europe indicated a total of 14,000 fatalities. The trend in mortality due to asthma in Europe has decreased by 64%. Furthermore, 212 million people worldwide are fighting COPD. It is considered the third most common cause of death globally, with over 3 million fatalities reported in 2019. The mortality rate caused by COPD worldwide has increased by 30% since 1990 [3]. Among the European population, the prevalence of COPD is 10%. COPD represents a mortality rate of 334,000 deaths in Europe annually, signifying 3.74% of total deaths. Since 1990, mortality in Europe due to COPD has increased by 12.5%. Additional respiratory afflictions, such as chronic bronchitis, lung cancer, and pneumonia, among others, contribute also to the burden. Tragically, in less-developed areas, many children under the age of 5 succumb to acute lower respiratory tract infections from various sources, including malaria, tuberculosis, or HIV [4]. The COVID-19 virus, as mentioned earlier, has also added to these health challenges [5]. According to Eurostat’s respiratory diseases statistics [6], in 2017, nearly 545 million people worldwide suffered from chronic respiratory diseases, marking a 40% increase since 1990. Despite a global rise in the number of individuals affected by chronic respiratory diseases during the period 1990-2017, the European population has experienced a modest decline. Unfortunately, the number of deaths worldwide caused by respiratory diseases remains high, with 379,000 deaths in Europe (accounting for 4.25% of total deaths) and almost 4 million deaths globally in the year 2019 (7% of total deaths) [3].

The diagnosis, detection, and treatment of respiratory diseases typically demand hospital resources, often involving costly and invasive methods. Commonly used methods like spirometry, pneumography, plethysmography, or capnography are typically invasive, often inconvenient for patients, and require expensive equipment primarily found in ICUs [7,8,9]. Monitoring the respiratory rate (RR) serves as a vital sign to track the progression of illness, and an abnormal RR is a significant indicator of serious health issues. Sufficient evidence supports the use of alterations in RR to predict potentially severe clinical events, such as cardiac arrest or admission to the intensive care unit [10,11,12,13]. These studies demonstrate that RR outperforms other vital measurements, including pulse and blood pressure, in discerning between stable patients and those at risk. By utilizing changes in RR measurements, patients at high risk could be identified up to 24 h before the event, achieving a specificity of 95% [14].

**Figure 1:**
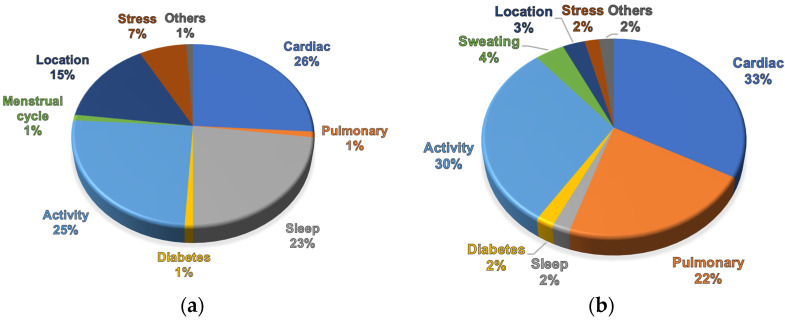
Respiration wearables statistics [19]: (**a**) marketed devices; (**b**) research works.

In response, research teams are directing their efforts toward the development of wearable and remote devices capable of continuous respiratory monitoring in diverse environments with a focus on natural physiological conditions. Various research domains, including sleep monitoring, breathing pattern analysis, and RR detection, call for convenient and wearable devices that patients can use at home for continuous monitoring and data storage [15]. Specifically tailored for respiratory activity monitoring, wearable and remote devices contribute to expanding medical care services, such as continuous air quality measurement, lung function monitoring [16], and sleep monitoring for detecting apnoea [17]. To enhance wearability across different activities, the current trend is also focused on exploring and developing smart fabrics [18]. Commercially available devices, as depicted in Figure 1a, indicate that only 1% of wearables actually integrate the detection of breathing-related events. A few may be also obscured within the cardiac and sleep area group, but the respiration in these areas is primarily only derived from heart rate variability (HRV). Contrastingly, pulmonary event detections, encompassing cough detections and respiration rate measurement, as depicted in Figure 1b, target 22% of all research [19]. This underlines the growing demand and anticipated surge in respiration sensors in wearable electronics.

Advancements in electronics design, especially battery technology, low-power embedded processors, and development of the software domain, which can give devices robust applications and more usable use cases, have enabled the development of new advanced wearables and remote systems.

**Figure 2:**
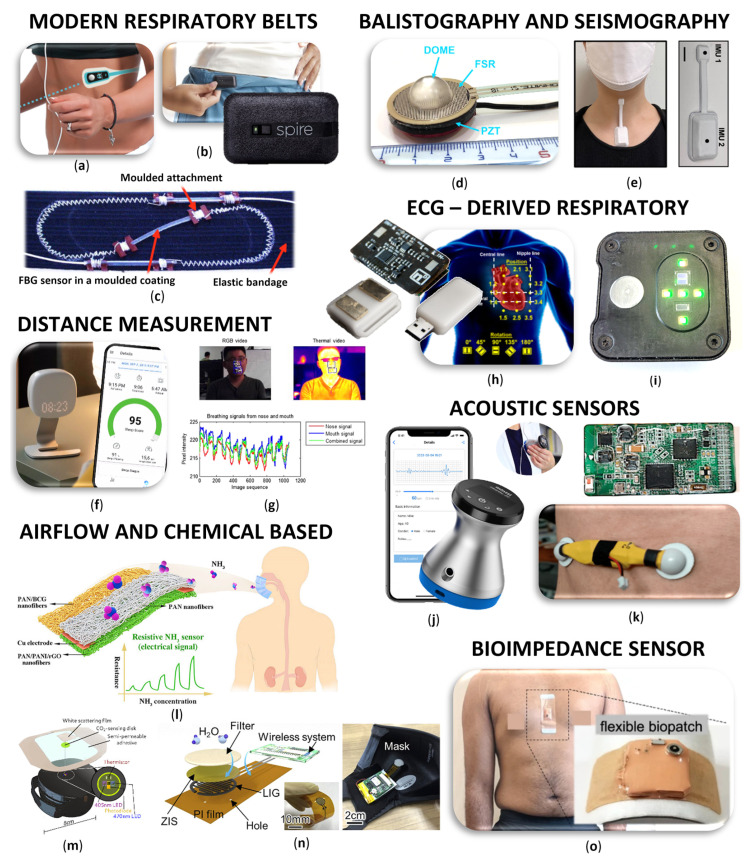
Wearable and remote respiratory sensors: (**a**) Resmetrix—chest strap powered by a proprietary sensor for monitoring breathing patterns, heart rate, temperature, activity, and position. Reprinted from ref. [20]; (**b**) Spire Health Tag—force respiratory tracker attached to clothing enhanced by physical activity and heart rate sensors. Reprinted from ref. [21]; (**c**) Respiration belt with embedded fiber optic sensors. Reprinted from ref. [22]; (**d**) Forcecardiography sensor for simultaneous monitoring of respiration, infrasonic cardiac vibrations, and heart sounds. Reprinted from ref. [23]; (**e**) soft skin-interfaced mechano-acoustic sensors for real-time monitoring and feedback on respiratory and swallowing biomechanics. Reprinted from ref. [24]; (**f**) Somnofy—ultra-low-power radar system for contactless analysis of RR and sleep stages [25]; (**g**) synergetic use of thermal and visible imaging techniques for contactless and unobtrusive breathing measurement. Reprinted from ref. [26]; (**h**) respiration derived from ECG amplitude-optimization of sensor placement. Reprinted from ref. [27]; (**i**) ePPG—own designed multisensor that calculates respiration from heart rate variability; (**j**) digital stethoscope Mintti Smartho-D2. Reprinted from ref. [28]; (**k**) acoustic and biopotential multi-sensor patch. Reprinted from ref. [29]; (**l**) dual-signal NH_3_ sensor for diagnosis of chronic kidney disease. Reprinted from ref. [30]; (**m**) continuous transcutaneous monitoring of CO_2_. Reprinted from ref. [31]; (**n**) flexible humidity sensor for sleep apnea monitoring. Reprinted from ref. [32]; (**o**) soft wearable flexible bioelectronics with bioimpedance measurement using ADS1292R (Texas Instruments, Dallas, TX, USA). Reprinted from ref. [33].

Our article delves into motivation and current trends. We explore technological advancements, challenges, and the potential impact of wearable and remote respiratory monitors on healthcare outcomes. As the field continues to evolve, the integration of wearable electronics for respiratory monitoring holds immense promise, shaping a future where individuals can seamlessly and intelligently manage their respiratory health in various life contexts. Our main goal is to summarize current knowledge, outline future trends, and provide scientists with a kind of springboard. A quick overview of the sensors that we will deal with is presented in Figure 2. Details are described in individual chapters.

## 2. Chest and Abdominal Movement Detection

One of the clever methods to detect respiration using wearable and remote electronics is the tracking of body movements caused by respiration (Table 1). The most well-known and, at the same time, the oldest approach entails monitoring the circumference or the volume of the chest using various forms of respiratory belts. Recently, with the incorporation of inertial measurement unit (IMU) sensors, it is also possible to monitor respiratory micromovements. Utilizing methods such as the seismocardiography, ballistography, etc., has gained popularity. The primary advantage lies in the cost-effectiveness of these sensors and their seamless integration into modern hardware designs. Over time, these sensors have transitioned from being attached to the human body to becoming integral components of everyday items that people interact with, including smart clothing, beds, chairs, and mattresses. Optical fiber-based sensors are also commonly embedded in these items. With the advent of modern bioamplifiers, deriving respiratory parameters from chest impedance variations has become also convenient. Remote methods include the extraction of chest movements from the visual signals using precise cameras or the deployment of Doppler radars. While these devices ultimately allow for monitoring of the temporal progression of the respiratory cycle and calculating RR, and in more advanced cases, respiratory volumes (RVs), they do not enable the determination of respiratory metabolism or the chemical and physical composition of exhaled gases.

### 2.1. Chest Belts and Their Modern Alternatives

Direct respiratory monitoring relies on the detection of humidity, pressure, and temperature of exhaled breath, but it is prone to environmental interference, leading to reduced accuracy and increased noise. On the other hand, indirect respiratory monitoring predominantly involves the use of strain or pressure sensors, typically employing stretchable electrodes or functional materials attached to the skin of the chest and abdomen [34].

The most basic and “oldest” chest belts for measuring respiration use resistance-based sensors. Each change in resistance values, caused by the expansion and contraction of the chest wall during breathing, corresponds to a detectable fluctuation in the current, registered by the sensor. This method is already very well known, so we present only a few pieces. The wearable Airgo belt [35], for example, utilizes a resistance-based system placed on the ribcage’s bottom, which detects chest circumference. The Airgo band, composed of stretchable materials with a silver-coated yarn, uses a microprocessor for data collection, Bluetooth for wireless communication, and inertial measurement for motion detection. A study involving 21 healthy subjects compared Airgo with a metabolic cart, focusing on key parameters.

A similar approach is used by piezoresistive sensors. In the context of respiration, piezoresistive sensors are often integrated into wearable devices or patches. One advantage of piezoresistive sensors is their ability to capture subtle variations in chest or abdominal movements with high sensitivity. Chu et al. [36] developed a wearable sensor to measure RR and RV simultaneously using low-powered piezoresistive sensors integrated with wireless Bluetooth units. The sensor, composed of a piezoresistive thin metal film, set in a silicone elastomer substrate, demonstrated high fidelity and linear responses. The sensors, with small footprints, were placed on the chest and abdomen and could record respiration signals even during walking and running. However, challenges include sensitivity to additional motions and artifacts, with proper sensor placement being crucial. Yuan et al. [37] developed a flexible piezoelectric sensor for respiratory monitoring based on thin-film polyvinylidene fluoride (PVDF) on fish lateral line structure. The monitoring system has been tested under various conditions, which provides a prerequisite for wide applications in daily health monitoring. Another interesting study on the use of a PVDF polymer was published by Lei et al. [38]. In this study, a piezoelectric PVDF film encapsulated in polydimethylsiloxane was used. The feasibility of using the suggested sensor patch for RR measurements was validated. The outcomes of these measurements showed no statistical differences compared to those obtained using a commercial respiratory effort transducer. In future wearable applications, piezoelectric sensors based on PVDF can be used in respiration monitoring for their high sensitivity, flexibility, and light weight.

Another measuring method, respiratory inductance plethysmography (RIP), is a non-invasive technique used to measure respiration by detecting changes in the inductance of coils or bands placed around the chest and abdomen. RIP is widely employed in wearable devices for continuous respiratory monitoring. The technology offers advantages like comfort, ease of use, and minimal interference with natural breathing, making it suitable for various applications, including sleep studies, physical activity monitoring, and respiratory health assessments. Wu et al. [39] proposed a wearable device that utilizes proven RIP technology, wireless body sensor networks, and accelerometers for sleep respiration monitoring. The textile RIP sensor, integrated into a suit for thorax or abdomen placement, incorporates a smart signal-processing algorithm for dynamic respiration rate extraction. Monaco et al. published a review that includes several studies of sensors working on resistive and RIP principles. The review is also supplemented by optical sensors and individual acoustic, electrical impedance, ECG-based sensors, etc., which we will discuss in detail in later chapters. [40].

Capacitive stretch sensors can also be used to measure respiration. They typically consist of a flexible material integrated with conductive elements. As the material stretches or contracts with the movement of the chest during breathing, the distance between the conductive elements changes. Whitlock et al. [41] proposed A-spiro, a wearable device for estimating respiratory flow, RV, and RR. Worn as a chest belt, it features a capacitive stretch sensor and IMU, transmitting data over Bluetooth. The sensor data processing involves three modules: data preprocessing for capacitance and accelerometer readings, breathing signal reconstruction using empirical mode analysis, and hysteresis modeling and flow estimation for predicting respiratory flow. Enokibori et al. [42] introduced Spiro Vest, an e-textile-based spirometer, which employs two length sensors on the chest and abdomen to detect torso expansion and contraction, calculating changes in torso and lung volume. The e-textile sensor features conductive fibers as warps and stretchable fibers as wefts. This textile can be expanded width-wise, altering the spacing between adjacent conductive warps. The changes in distance lead to electronic capacitance variations among the warps, which the device transforms into length changes in the sensor. Park et al. [43] integrated a flexible capacitive pressure sensor into a waist belt for detecting real-time respiration signals through capacitive transduction. The sensor consists of polydimethylsiloxane-based silver nanowires and carbon fibers, which demonstrated high sensitivity (0.161 kPa^−1^), a wide working range (up to 200 kPa), and durability (over 6000 cycles). Signal processing involved a low-pass filter and MATLAB-coded finite impulse response (FIR) filter to remove noise. Despite some voltage peaks during upper body movement, the wearable sensor proved effective for non-contact real-time respiration monitoring.

The rising field of self-powered sensing methods has brought significant attention to wearable technology in recent years. One of the most promising technologies in this area is triboelectric nanogenerators. Li et al. [34] employed a lightweight retractable self-powered sensor (RSPS), which incorporates a miniature rotating thin-film triboelectric nanogenerator (RTF-TENG) capable of enduring over 1 million stretching cycles. The RSPS is part of a wireless multifunctional wearable respiratory monitoring system, facilitating daily portable monitoring of various respiratory parameters. This system includes an adjustable nylon strap and integrates a Wi-Fi module, STM32 (STMicroelectronics, Geneve, Switzerland) controller, analog-to-digital (AD) acquisition module, and charge amplifier for wireless communication. The RTF-TENG, the key component, produces AC signals during stretching.

There are various commercially accessible medical devices used for monitoring respiration, equipped with proprietary sensors, designed to be user-friendly for both clinical and home settings. Resmetrix [20] introduces a novel wearable system employing a chest strap to continuously monitor breathing patterns, vital signs, and disease progression. The wireless connection to a smartphone app enables real-time monitoring and detection of respiratory issues. Proprietary sensors with an AI-powered algorithm can effectively detect changes in respiratory patterns during a mild asthma attack and monitor respiratory signals during exercise. Spire Health [21,44] has introduced the commercially available Health Tag (Spire, Inc. San Francisco, CA, USA) respiratory tracker, designed to predict exacerbations and hospitalizations in severe COPD patients. With sensors attached to clothing, it offers a set-it-and-forget-it approach, recording respiratory data, physical activity, HR, and sleep. The proprietary respiration force sensor measures respiratory effort, the expansion and contraction of the thoracic cavity and lower abdomen, to provide a highly detailed view of the full respiratory waveform. The machine-washable, dryer-safe device is specifically designed for elderly individuals. Fourth Frontier offers another interesting commercial device called Frontier X2 (Fourth Frontier Technologies Ltd., London, UK) [45], which is a wearable chest band optimized for heart rate and respiration rate monitoring during exercise or sleep.

Different methods can be employed to sense chest movement, and as an example, we can also cite an alternative where the change in the circumference of the chest affects the gain of the textile antenna. Wagih et al. [46] proposed an e-textile sensor that utilizes a broadband monopole antenna with high gain and efficiency, fabricated using conductive fabric attached to a textile substrate. Experimental characterization of the sensor demonstrates accurate breath detection.

Chest straps are primarily designed for recording RR or RV and are known for their high accuracy in measuring these parameters compared to alternative wearables. This precision is thanks to their proximity to the chest or abdomen, where respiratory movements are more notable. For simple applications, they can also be placed over clothing. To achieve the highest accuracy, it is recommended to utilize dual breathing belts, capturing both chest and abdominal breathing. Chest belts offer great stability, particularly during physical activities, which makes them well-suited for sports and fitness applications where body movements are dynamic. Their proficiency in continuous monitoring allows a comprehensive perspective on respiratory patterns throughout the day and during specific activities. However, some users may feel chest belts are less comfortable, especially during prolonged wear, and tight straps might induce discomfort or affect natural breathing movements. Ongoing research aims to address comfort problems by transforming sensors into patch formats. Particularly suitable for this task are piezoresistive sensors. The drawback is that these patches need closer contact with the body’s surface and demand a more precise selection of the body location. It is excellent that chest belts outshine other wearables in battery life. Their focused functionality, primarily centered on respiration monitoring, contributes to extended battery life. Additionally, the dynamics of respiration impose less demand on sampling frequencies, further contributing to prolonged battery life.

### 2.2. Seismocardiography, Ballistocardiography, and Similar Methods

Seismocardiography (SCG), introduced in 1961 by Bozhenko [47], involves the recording of body micro-vibrations produced by the cardiovascular and respiratory systems at the thorax [48,49,50,51,52]. This method shares fundamental principles with technologies such as forcecardiography, gyrocardiography, vibrational cardiography, kinocardiography, etc. The SCG signal continuously reflects the activity of both the cardiovascular and respiration systems. SCG components are presented in all three axes of the accelerometer, with each axis revealing a distinct pattern [53]. The low-frequency component matches the chest wall motion induced by respiration and a high-frequency component matches the heartbeat. [54]. The SCG signal is affected by respiration in three ways: first, by shifting the SCG baseline due to the chest wall movement; second, by SCG amplitude variation caused by changes in pressure inside the chest; and third, through respiratory sinus arrhythmia in HRV [55]. Current research teams are either working on proposing extraction algorithms [56] or enhancing the quality through quantitative analysis of motion artifacts [57,58]. This involves utilizing an adaptive recursive least-squares filter [59] or time–frequency distribution analysis [60] or employing two cooperating accelerometers [54,61]. Using SCG, we can detect mechanical aortic opening, aortic closure, the point of maximal blood acceleration in the aorta, opening of the mitral valve, closure of the mitral valve, rapid filling of the left ventricle, accelerometer-derived respiration, RR, HR, and respiration amplitude.

Another relevant method is ballistocardiography (BCG), introduced by Gordon in 1877. This technique captures the motions of the entire body induced by blood flow during cardiac contractions [62]. Subsequent researchers, such as Henderson [63], Heald and Tucker [64], Starr and Krumbhaar [65], and Rubenstein [66], among others, have explored the influence of respiration on the BCG signal. Unlike SCG, micro-motion is not limited to the chest but extends to the entire body surface [67]. The BCG component also encompasses movement along all three axes and can be measured as acceleration, velocity, displacement, or force signal [53]. While the impact of respiration on ECG [68] has been extensively studied, research on the effects of respiration on SCG and BCG lags. According to a systematic review by Han et al. [69], only a small percentage of studies focus on respiratory monitoring [67]. BCG is primarily influenced by cyclical changes in intrathoracic pressure resulting from pulmonary ventilation [18], serving as a source of respiratory-cardiovascular coupling [70]. Pulmonary stretch receptors stimulated during inspiration activate the Hering–Breuer reflex of motoneurons in the nucleus accumbens [71]. The thoraco-abdominal respiratory pump, in turn, generates oscillations in venous return and left ventricular afterload, leading to arterial pressure oscillations [71,72,73], consequently triggering the baroreflex. Additionally, there is a direct interaction between the brainstem respiratory oscillator and cardiac vagal preganglionic neurons in the nucleus accumbens [67,70]. The latest literature reflecta a significant enthusiasm for employing these techniques in extracting respiratory data. The potential for concurrent monitoring of respiratory and cardiovascular signals using BCG or SCG opens avenues for observing vital physiological functions during routine activities outside the hospital setting, such as demanding sports, mental tasks, sleep, etc. A notable advantage of SCG and BCG techniques in respiration detection lies in their cost-effectiveness and straightforward implementation. However, akin to several other methods, a notable drawback is the lack of information about the composition of respiratory gases.

If we now move on to specific products currently available, we can start with a typical representative of SCG monitoring, a MagIC vest system from Marco Di Rienzo et al. [55,74]. This system presents a modified version of a textile-based wearable device designed for inconspicuous recording of ECG, respiration, and accelerometric data to evaluate sternal SCG in daily life. Their research not only delves into signal processing but also includes a comprehensive comparison with alternative methods like phonocardiography and BCG. Tadi et al. [75] conducted intriguing SCG research on extracting respiratory and heart activity from accelerometer signals using Freescale MMA8451Q accelerometer (Freescale Semiconductor, Austin, TX, USA). They compared extracted parameters not only with traditional ECG and chest belts but even with CT images, showcasing a thorough exploration of the method’s capabilities. In the realm of forcecardiography, an article [23] discussed a sensor utilizing a force-sensing resistor (FSR03CE, Ohmite Mfg Co., Warrenville, IL, USA) equipped with a dome-shaped mechanical coupler. The authors conducted a detailed analysis and compared results with ECG-derived respiration and a resistive respiration band in an experiment involving seven participants. Additionally, research focusing on the analysis of respiration in individuals undergoing thoracic surgery using a smart IMU sensor is another noteworthy contribution to this field, with potential applications in home environments as well [76]. Respiration monitors find extensive use in the realm of biofeedback. From a technical standpoint, these devices are often straightforward, focusing on user-friendliness and aesthetic design. Nevertheless, they prove to be effective tools. Notable examples include the iBreve brooch (iBreve, Dublin, Ireland) [77], ingeniously attached to the hem of a bra, and the Oxa (Oxa Life, Rumlang, Switzerland) [78], designed for placement on the chest during relaxation exercises. In the domain of commercially available BCG devices, Sadat-Mohammadi et al. [79] presented a wearable respiration sensor based on an accelerometric sensor and random forest classifier and achieved an accuracy of up to 93.4% while being less sensitive to body and sensor movement artifacts. In the article by Tavakolian et al. [80], the focus was on enhancing BCG processing through the incorporation of respiration information. The study involved experiments conducted on 45 subjects, encompassing individuals both with and without heart-related pathologies. The comprehensive data acquisition process included the measurement of BCG, 12-lead ECG, pulse oximetry, respiration, and heart sounds. All signals were meticulously acquired using a Biopac biological data acquisition system [81]. In the study conducted by Pandia et al. [82], the primary focus was on frequency domain analysis of respiratory variations within the SCG. To achieve this, they utilized a miniature MEMS (microelectromechanical system) accelerometer (LIS3L02AL, STMicroelectronics, Geneva, Switzerland) and conducted their research on 18 healthy volunteers. The researchers divided the entire SCG signal bandwidth (0–100 Hz) into 5 and 10 Hz frequency bands. They systematically compared the spectral energies observed during inspiration, expiration, and apnea. Notably, statistically significant differences were identified within the 10-40 Hz frequency range. This approach provided valuable insights into the respiratory dynamics captured by the SCG signal. Kang et al. [24] contributed significantly with an outstanding article, showcasing the practical implications in the realm of wearable electronics. They engineered soft skin-interfaced mechano-acoustic sensors for real-time monitoring and patient feedback on respiratory and swallowing biomechanics. Their research involved rigorous validation studies on a cohort of 67 healthy adults and 4 patients with dysphagia, comparing the results to existing clinical standard equipment. Remarkably, the authors also assessed the differential mode of operation, demonstrating comparable performance even during routine daily activities and intense exercise. The neck patch itself features two IMU units, enabling complete communication and signal analysis. Additionally, they developed a variant incorporating a haptic sensor. The work conducted by Da He et al. [83] is noteworthy for its originality in exploring the side behind the ear as a potential location for wearable vital signs monitoring. In their research, the authors specifically focused on photoplethysmography (PPG) and BCG. For BCG measurements, they employed a 25 mm × 25 mm hybrid sensor with two capacitive electrodes for differential sensing and one dry electrode for common-mode feedback, connected to a high-impedance LMC6064 (Texas Instruments, Dallas, TX, USA) operational amplifier. This BCG sensor not only provided continuous HR and RR monitoring but also demonstrated correlations to cardiac output and blood pressure. The use of the unique location behind the ear adds a novel dimension to wearable vital sign monitoring. Morra et al. conducted a study titled “Influence of sympathetic activation on myocardial contractility measured with BCG and SCG during sustained end-expiratory apnoea” on 28 healthy men [84]. In this research, they measured BCG and SCG at a sampling rate of 50 Hz using two detectors, each comprising a 3D accelerometer and 3D gyroscope. One detector was placed on the manubrium of the sternum below the clavicle over the superior mediastinum, while the second was positioned in the lumbar lordosis curve, between the second and third lumbar vertebrae. Simultaneously, one-lead ECG, blood pressure, SpO_2_, and end-tidal CO_2_ were also measured. Using this hardware setup, Morra et al. further conducted BCG and SCG detection to assess hemodynamic changes during simulated obstructive apnea on 20 volunteers [85]. These studies contribute valuable insights into the influence of sympathetic activation on myocardial contractility and the hemodynamic effects of simulated obstructive apnoea. Some research even focuses on recording the movement of specific muscles active during respiration such as intercostals and diaphragm. Uduak et al. [29] designed a device using commercial EMG patches combined with a piezoelectric microphone for monitoring intercostals and diaphragm movement and breathing sound acquisition.

The realm of BCG research offers intriguing alternatives beyond directly measuring signals on the body, utilizing everyday objects with which people closely interact. Various sensors embedded in beds, mattresses, and chairs show promising potential. A typical BCG sensor is the Emfit QS Active (Emfit Ltd, Vaajakoski, Finland) sleep monitor [86]. Placed beneath the mattress, it meticulously records and assesses crucial sleep parameters. This innovative device measures HR, HRV, breathing cycles, sleep cycles, movements in bed, overall recovery, stress levels, snoring, and, above all, sleep quality. Vehkaoja et al. [87] made a significant contribution by investigating the effects of sensor type and location on signal quality in bed-mounted BCG, HR, and RR monitoring. They employed force sensors made of piezoelectric PVDF film and electret polymer material placed under the mattress topper, conducting experiments in 23 different positions. Brüser et al. [88] introduced an interesting solution for measuring HR, RR, and complex breathing patterns using a BCG sensor based on a multi-channel optical sensor array consisting of two infrared LEDs (SFH4250, OSRAM, Munich, Germany) and a photodiode (BPW34FAS, OSRAM, Munich, Germany). Dynamic forces acting on the mattress surface caused deformations that modulated the light intensity sensed by the photodiode. Static-charge-sensitive beds [89,90] offer a simple and inexpensive BCG alternative, enabling continuous long-term monitoring of HR, RR, respiratory amplitude, and body movements. Albukhari et al. [91] introduced bed-embedded HR and RR detection using longitudinal BCG and pattern recognition. They employed a low-cost, off-the-shelf load cell installed on a typical hospital bed with a machine-learning algorithm. Mack et al. [92] used two pressure pads installed on a mattress for BCG evaluation in a sleep-monitoring system. They assessed HR and RR in 40 healthy subjects, complementing the study with traditional polysomnography. Zhao et al. [93] used a set of oil pressure sensors embedded in a micromovement-sensitive mattress to identify sleep apnea syndrome. Using HR and RR signals from 42 subjects over 3 nights, they implemented a knowledge-based support vector machine (KSVM) classification model. In the study by Lee et al. [94], four load cells (CBCL-6L, Curiosity Technology, Pajusi, Gyeonggido, Republic of Korea) were installed below the plane of the baby bed, connected to a Wheatstone bridge configuration. This configuration produces an electrical signal in response to force changes induced by cardiac activity and respiratory movements. The authors developed algorithms for determining HR and RR as part of their work. Cimr et al. [95] present the application of a mechanical trigger for the unobtrusive detection of respiratory disorders based on body recoil micromovements. They used four tensimeters installed on the legs of the bed to detect 3D micromovements. Signal analysis was based on a Convolutional Neural Network (CNN) with 12-dimension information obtained from 20 volunteers. Martin-Yebra et al. [96] evaluated respiratory- and postural-induced changes in the BCG signal using a multicomponent biomechanical force plate, based on 3D piezoelectric load cells (Type 9286B, Kistler^®^, Wien, Austria, 600 × 400 mm). Time-warping averaging was employed on a group of 20 healthy volunteers. From our own experiments, we can mention our pressure measurement using a Treston DMP 331 sensor in a seat air cushion from 2011 [97], where we used microsensors to evaluate cognitive processes. From the pressure sensor, we determined HR and RR while driving in the car simulator.

The narrative review by Balali et al. [67] provides an excellent and comprehensive overview of recent advancements in extracting respiratory signals, computing cardiorespiratory interaction markers, and exploring practical applications, including the investigation of sleep-breathing disorders. The authors delve into the investigation of cardiorespiratory interaction using SCG and BCG. They highlight the growing trend towards incorporating artificial intelligence and the associated benefits in real-world scenarios outside of clinical settings, emphasizing reductions in costs and time. The review also includes a comparison of different sensors utilized for these applications.

SCG and BCG sensors are primarily designed for recording RR and less for RV. These sensors operate on a nonintrusive principle by detecting vibrations resulting from both respiration and the heartbeat. This dual functionality provides a comprehensive perspective on cardio-respiratory dynamics. In comparison, SCG sensors are used more in the form of a patch, and versatile BCG sensors are used in wearable devices in a wider range, for example, in smartwatches, personal belongings, or bed linen, and even in this way, they record physiological parameters without the need for direct contact with the skin of the person being measured. Such a measurement is very comfortable. However, it must be recognized that the closer the sensors are to the body, the more accurate the measurement is, and from this point of view, SCGs are considered more accurate. Since the SCG also commonly uses an accelerometer on the chest, the position of the body is also recorded well, which can be used to filter out signal interference by body movements. Overall, vibration from body movements and interference from nearby devices are considered disadvantages of both sensors. Especially with BCG sensors, extracting respiratory information is a big challenge and requires advanced signal-processing techniques for accurate interpretation. For example, the complexity of such an operation may require additional computing power associated with higher sampling rates and, thus, have much higher energy consumption requirements.

### 2.3. Chest Impedance Measurement—Bioamplifiers

Measuring body impedance to determine respiration involves assessing the electrical impedance of the body, particularly the thoracic region, to derive information about the respiratory process. Analyzing these changes provides valuable insights into respiratory parameters such as rate and volume. This non-invasive method offers potential applications in wearable devices for continuous respiratory monitoring.

Järvelä et al. [98] introduced a wearable sensor, which utilizes three electrodes to measure thoracic impedance variations, enabling accurate RR monitoring. The wireless sensor connects to a mobile monitoring device, analyzing signals and transmitting data to a central station. RR determination relies on evaluating thoracic impedance changes using a “dual vector” approach. A comparison with capnography was performed using Bland–Altman analysis, with error grid analysis assessing risk levels. The study, involving 40 adult ward patients, demonstrated the reliability of RR measurements with the new sensor. Fedotov et al. [99] developed a wearable respiratory monitoring device, which employs bioelectrical impedance plethysmography and a 3D accelerometer to capture respiration and body movement. To mitigate motion artifacts, a combined hardware and software solution was created using a band-pass filter and adaptive noise cancellation software implemented in the microcontroller’s firmware. Khan et al. [100] compared electrical impedance plethysmography (EIP) with spirometry. The objective was accurate spirometer output estimation using EIP channel outputs. They employed 10 EIP channels placed at various torso locations. A learning algorithm was trained and tested on non-overlapping data subsets, comparing the estimated spirometer signal with the reference signal. A novel Segregated Envelope and Carrier estimation approach was introduced, based on amplitude-modulated (AM) signal approximation, separating amplitude and breathing rate components. The proposed virtual spirometry framework combines multichannel EIP and bio-signal processing, demonstrating efficacy in spirometer output estimation. Motion artifact detection, support vector machine (SVM) regression, and time–frequency signatures of motion artifacts in EIP channels offer promising avenues for physical activity monitoring.

As part of the trend, leading integrated circuit manufacturers are incorporating EIP directly into their biopotential transducers. The circuit series ADS129xR (Texas Instruments, Dallas, TX, USA) is especially popular [101]. It is a low-power, 8-channel, 24-bit analog front end (AFE) with fully integrated respiration impedance measurement. Texas Instruments also offers other components like AFE4960 [102] and AFE4500 [103], providing a comprehensive solution for measuring bioimpedance (BioZ), including respiration monitoring, with a 22-bit resolution and I^2^C and SPI interfaces. Analog Devices offers the ADAS1000 (Analog Devices, Wilmington, MA, USA) [104], a low-power ECG AFE with five acquisition channels, AC and DC lead-off detection, and thoracic impedance measurement. Another example component from Analog Devices is the MAX30001 [105], which operates with ultra-low power and integrates a single-channel biopotential AFE with bioimpedance for respiration measurement. The AS7058 (OSRAM, Munich, Germany) [106] features two 20-bit ADCs for PPG acquisition and one 20-bit ADC for ECG/BioZ acquisition, capable of measuring respiration. A good example of the application of these bioimpedance sensors is the soft flexible bioelectronic system by Kim et al. [33], who applied it to calculate metabolic costs and physical effort.

Sensors based on chest impedance are considered nonintrusive but need direct contact with the skin of the chest. They can therefore appear uncomfortable to wear, even though the forms of wear nowadays try to think about the wearer’s comfort as well. The difference in signal quality can be affected by humidity, skin condition, fit, and other factors. They detect changes in the electrical impedance of the chest caused by breathing movements of the body and can continuously and unobtrusively record not only RR but also RV. They are well integrated into ECG holsters, chest patches, smart clothing, and other various wearable devices. Chest impedance-based sensors can operate at various sampling frequencies, which contribute to energy efficiency and therefore lower energy consumption. All things considered, accurate measurement can be ensured by proper contact and adjustment. Of course, interference with body movements can negatively affect the quality of the signal, but post-processing of the signal can improve the accuracy of respiratory data.

### 2.4. Optical Fibers

Optical sensors, especially with Fiber Bragg Grating (FBG), are very important in the development of medical devices for measuring physiological parameters [107,108,109,110]. They offer a non-invasive approach with interesting metrological properties and electrical safety, they are cheap and resistant to water and corrosion, and, due to their immunity to strong magnetic fields, they can also be used in MRI or CT testing [110]. When monitoring respiration during exercise, it is possible to sweat easily and this will not affect the measurement, which can be a problem with other materials [111].

Optical fibers are made of high-quality glass or platinum materials that transmit signals without major losses. They are thin, light, transparent, and easily incorporated into elastic bands or textiles [112], and are therefore very comfortable to wear [113]. They can also be directly incorporated into pillows, mattresses, or patient beds in a hospital environment [114,115]. However, they are hard and fragile, which is why they are encased in various protective materials (lit). The principle of measurement is the periodic change in the refractive index along the optical fiber, and when breathing, lifting the area of the chest and abdomen and physical movement of the shoulder are converted into a shift in wavelength. During breathing, a spectral change in the reflected/transmitted radiation through the Bragg grating is recorded due to changes in its geometric parameters due to external forces acting on it [112].

The advantage of FBG optical fibers is the sensitivity of the probe only in the place of the optical grid, while they are not sensitive to vibrations and other unwanted effects. The sensor system can consist of a whole series of FBG sensors that can be used to monitor different physiological variables at the same time without mutual interaction through their shift in the spectral region. However, the disadvantage is the more demanding evaluation of the output signal, which can increase the price of such a system. Better signal detection is also needed, which has lower signal-to-noise ratios. For example, Witt et al. [22] focused on the development of medical textiles with FBG optical fibers for measuring RR in the MRI environment where they also tested the systems, which has an advantage in that it also offers an analysis of the patient’s breathing cycle. The goal of the study of Ciocchetti et al. [116] was to create a smart textile using two FBGs for continuous breathing monitoring. The authors also performed a previous biomechanical analysis of chest-wall movements during breathing, which they used to develop their device. In another article, Lo Presti et al. [107] focused on the fabrication of a flexible sensor based on FBG encapsulated in silicone rubber and De Jonckheere et al. [117] described the use of FBG sensors in textiles with a focus on anesthetized patients who are to be examined under MRI.

In addition to the fiber-optic system based on an FBG, the authors also described the use of a system based on a fiber-optic interferometer (FOI), where interferometric probes show slightly better measurement accuracy in the case of recording respiration [109]. The interferometric principle, unlike the measurement of the wavelength of reflected optical radiation, uses the measurement of the phase difference between two waves. It is an even more sensitive principle and managed to provide a higher accuracy of 0.13% in the case of respiration measurements. The advantage of the interferometric system is that it creates an amplitude modulation at the output that can be detected by ordinary photodetectors. Overall, the entire system can be constructed from commonly available components, which means that it is somewhat more cost-effective. Among the disadvantages, it is necessary to mention the long optical fiber (even meters), which can be a problem for certain applications. It is also more sensitive to vibrations and is not so suitable for multisensory use. Each sensor needs its own fiber and detection components.

Bennett et al. [118] used a multimode optical sensor for sensing respiration in the chest, which can be incorporated into fabrics. This device is still in the testing phase. Multimode optical fibers (MMFs) support multiple transversely guided modes for a given optical frequency and polarization. They can guide light with low beam quality and high optical performance. The principle is based on the theory of the micro-bending of optical fibers, where the fiber is deformed during respiration, the light is modulated in the MMF, and the camera records these changes. MMFs are often affected by external noise, and improving the performance of the sensor in the future will allow signal filtering and algorithm development. The detection of RR using MMF was also mentioned in other works, where the fiber was embedded not only in textiles but also in mattresses or mattresses. [113,119,120]. Likewise, Zha et al. [121] in their work focused on multimode optical fibers. In this case, they used two thermoset multimode silica optical fibers with an elastomer optical fiber incorporated into the respiration-monitoring belt. The sensor was tested by 10 volunteers with a maximum error of 1 bpm for RR.

Nasr et al. [122] used a fiber optical BCG sensor built into a chair back. They used two spectral-based approaches (unsupervised classification based on the Gaussian Mixture Model and supervised classification based on K-Nearest Neighbors) to evaluate HR and RR, achieving an accuracy of 94.6%.

Plastic optical fibers (POF) contain a respiration sensing part, a light source, and a photodiode deamplifier system. The intensity of the reflected light changes due to the respiratory movements of the chest and, thus, the distance between the mirror and the distal end of the POV changes and these changes are recorded, and the RR is obtained [123,124]. The POF breathing sensor in the form of a textile belt was presented by Krehel et al. [125]. Wang et al. [126] in their work described a plastic optical fiber (POF) sensor in the shape of the letter D. The advantage is its high sensitivity, low cost, and use in various conditions and physiological states. The sensor was built into the elastic waist where it reacts to the bend caused by the movement of the chest during breathing. In another study, Han et al. [127] investigated POF pressure sensors embedded into mattresses. Such a smart mattress can record breathing with an absolute error of less than one breath per minute. Also, Sartiano et al. [128] focused on a low-cost POF pressure sensor built into the mattress. They designed a multi-point sensor capable of sensing respiration. In the work of Xu et al. [129], the authors describe a grooved POF sensor embedded in polyurethane that can monitor a person’s RR. In their studies, they investigated the optimal placement of the sensor train and the sensitivity to tension and presented the entire sensor device in the form of a belt. The device showed higher accuracy in sitting and standing than in various other postures.

Which system is better is difficult to say, as it depends on the specific application and use. All of them have the advantage of the possibility of remote evaluation, which, nowadays, is necessary for the development of sensory wearable devices. Sensors made of optical fibers are characterized by sensitivity to changes in physical parameters, which allows accurate detection of fine respiratory thorax (RR and RV). The advantage is that they are non-disruptive, resistant to electromagnetic interference, can be integrated into various forms of devices, textiles, chest patches, and furniture, and in general, can be inserted into very convenient forms of wearable devices. They are very suitable and often used in various environments due to their properties, such as in MRI. Thanks to their resistance to interference, they are also suitable for measuring other additional physiological parameters than just respiration and are suitable for use in multisensory devices. However, their disadvantage remains the need to use sophisticated signal-processing techniques and advanced algorithms for accurate interpretation due to the fact that they generate complex signals. Compared to other sensors, the application of optical sensors can be more expensive because of this, and therefore, the devices that use these sensors can be at a higher price level. The relative fragility of optical fibers is also a disadvantage. Optical sensors can achieve high sampling rates, enabling a detailed temporal resolution in breathing monitoring and complex analysis of respiratory patterns. Energy consumption can be different depending on specific optical sensors, but the implementation of FBG sensors in wearable devices shows moderate energy consumption requirements.

### 2.5. Radar Systems

True noncontact respiration measuring can be achieved using radars. The advantage of this approach is that using advanced algorithms and filtrations, they can process respiration even from a greater distance. They can often be easily mounted on walls and in elevators, beds, alarm clocks, etc.

Greneker [130] introduced one of the first noncontact respiration rate monitoring systems known as the Radar Vital Signs Monitor (RVSM). Initially designed for monitoring Olympic athletes at distances exceeding 10 m, the RVSM utilized the Doppler phenomenon to detect chest movements induced by breathing. Kukkapalli et al. [131] presented a wireless micro radar system for continuous breathing monitoring, which measures the relative motion between the radar and chest wall for the estimation of respiration patterns. The radar, operating at 24 GHz, employs a custom analog narrow-band amplifier circuit for reducing environmental noise. Data collected from the radar are transmitted via Wi-Fi to a laptop and processed using a moving average filter and Fourier transform for RR extraction. The system, designed as a wearable neck pendant, was compared to a commercial respiratory monitoring sensor as a gold standard. Experimental results demonstrated over 95% accuracy in predicting breathing rates across ten subjects. Xia et al. [132] used a stationary radar for detecting the radar displacement signal of heartbeat (RDH). The RDH signal, derived through complex Fourier transform and band-pass filtering of the radar signal, facilitated the detection of fiducial points such as aortic valve opening (AO). The evaluation of 22 subjects demonstrated that AOs detected by RDH achieved an average detection ratio of 90%, indicating a high correlation with AOs detected by the ECG R-wave. This setup, with signal-processing changes, would be suitable for RR monitoring as well. Guohua et al. [133] present a non-contact method using radar to monitor both the heart rate and RR of human subjects. The system uses radar to detect the ballistocardiogram (BCG) signal, which is analyzed in the time and frequency domains, along with ECG signals measured by electrocardiography. Wavelet transform, specifically using the Symmlet mother wavelet of order 8, was employed for simultaneous information in the time and frequency domains. The study by Sharma et al. [134] introduced a wearable radio-frequency sensor utilizing near-field coherent sensing (NCS) on 20 healthy participants performing voluntary breathing exercises in various postures. NCS, a non-invasive technique, involves transmitting a low-power continuous-wave RF signal into the body using over-clothing antennas, allowing direct measurement of heart, lung, and diaphragm motion. Two prototype sensors were strategically placed: one near the heart and the other below the xiphoid process. The study focuses on accurately estimating parameters such as RR, RV, and HR. A very practical device is presented by Somnofy [25]. It is a bedside device that uses ultra-low power radio waves to detect body movement and AI for detailed analysis of sleep stages and RR. The device has undergone clinical validation against PSG.

Radar sensors and sensors with the Doppler effect represent an innovative remote and non-contact approach. Non-contact can be a great advantage in specific cases where it is not possible to use sensors with skin contact. Radars also offer flexibility in placement. These sensors are used for integration into devices of various shapes such as alarm clocks, or they can be placed on the ceiling and, thus, offer wide flexibility in their use. The sensors can provide real-time monitoring of breathing patterns and thus detect respiratory anomalies. Due to non-contact, however, there is the disadvantage of external interference, which is higher than with non-contact devices, as well as the disadvantage of complex signal processing. A robust signal filtering mechanism is required to obtain the desired signal, which results in higher energy consumption.

### 2.6. Camera Systems

Estimating respiration from videos involves capturing and analyzing body movements to determine the individual’s RR. Video-based methods can provide another non-contact and remote approach for monitoring respiration.

Chen et al. [135] presented a framework for remote RR monitoring using face videos. The proposed framework employs motion compensation, two-phase temporal filtering, and signal pruning for accurate RR results and simultaneous measurement of HR and HRV. The system uses a two-phase temporal filter with an Infinite Impulse Response (IIR) filter for signal enhancement. The Lomb–Scargle periodogram was applied for spectral analysis of detrended HRV to estimate RR. The experimental results demonstrate the feasibility and effectiveness of the method for RR estimation in rest cases. Challenges included addressing poor signal quality in pulse signal extraction, especially concerning voluntary motions, expressions, and illumination changes. Stubbe et al. [136] created the Opto-Electronic Plethysmography (OEP) system for breathing rates and compared measured signals with a spirometer. Twelve retro-reflexive markers were strategically placed on the chest to capture respiratory movements accurately, using eight infrared cameras. OEP processing involved calculating chest volume based on marker positions and Fast Fourier Transform. Statistical analysis demonstrated strong agreement between OEP and spirometer-derived breathing rates, supported by high-amplitude values in the frequency domain. Correlation coefficients and Bland–Altman plots affirmed the accuracy and reliability of OEP-based estimations in comparison with spirometer measurements. Zhu et al. [137] published an RR monitoring technique based on infrared imaging. They developed a tracking algorithm capable of tracing facial features associated with respiration. These features, manually selected from the initial frame in the video, involved three specified windows. Two windows covered the periorbital regions, spanning from the bridge of the nose to the inner corner of the eyes, representing the warmest facial areas. The third window, positioned at the apex of the nose, represented the coolest facial area. Their algorithm successfully tracked these windows across subsequent images, and the respiration signal was derived from a region beneath the nose. Chekmenev et al. [138] opted for a thermal camera with a focal plane array designed for long-wave infrared sensing. They conducted body temperature measurements in specific areas, including the neck region, carotid vessel complex, and nasal region, with manual selection of these regions. The researchers developed a wavelet analysis technique to extract both the ECG and RR. In a study conducted by Al-Khalidi et al. [14], a thermal camera was employed to observe variations in skin surface temperature related to respiration. Segmentation of the images was performed, followed by the application of an algorithm to identify and track a circular area centered on the tip of the nose. This selected area was divided into eight equal concentric segments, and the pixel values within each segment were averaged to derive a singular value representing the skin temperature in that segment. This process was repeated for each image, and plots of average temperature against time for the segments were generated, illustrating the respiration signal associated with each segment. Prochadzka et al. [139] analyzed breathing using thermal and depth imaging camera video records. Their goal was to use these video records as alternative diagnostics of breathing disorders in the home environment. The methods include specific image processing algorithms, computational intelligence tools, digital filters, and spectral estimation tools. Mutlu et al. [140] monitored respiration phases using infrared thermography without image segmentation. They used the thermal camera FLIR A325sc (FLIR Systems Co., Shatin, Hong Kong) with a 50 μm lens, 60 fps, and a 320 × 240 pixel resolution.

A brief overview of the use of thermal cameras for respiration detection was conducted by Lewis et al. [141].

Similar to radars, cameras (conventional or thermal) can be used for remote non-contact monitoring. They enable unobtrusive monitoring of breathing (mainly RR) based on sensing visible movements of the chest or capturing thermal patterns of breathing. Cameras can be implemented in the home environment, hospitals, medical facilities, or public spaces. The monitoring of premature babies is also very interesting, where non-contact is very welcome.

The disadvantage can be low-light conditions, where poor light can affect the accuracy of RR sensing during breathing movements, but when sensing temperature patterns, light does not play such an important role and this method is more suitable in low light or in the dark. With camera recording, the privacy of recorded persons may be questionable, but when cameras only record temperature patterns, this question is not such a big problem.

The disadvantage of thermal cameras is their higher price and lower spatial resolution, which limits the detailed analysis of breathing patterns. Also, the power consumption and software requirements for algorithms are higher for cameras than for other respiratory sensors. The cameras are versatile, they can monitor continuously, and interestingly, they allow the detection of breathing in several people at the same time, which other sensors normally do not provide.

**Table 1:** Chest and abdominal movement-based respiratory monitoring.

Sensor Type	Application	Sensing Element	Key Parameters	Ref.
Chest belt	Respiration	Resistance based	BLE ^1^, IMU ^2^, Motion detection	[35]
Chest belt	RR ^3^, HR ^4^, HRV ^5^, activity	Proprietary sensor	Bluetooth, Mobile app, 24 h working time	[45]
Patch on chest and abdomen	RR, RV ^6^	Piezoresistive sensors	Bluetooth, Small footprint, Linear response	[36]
Band over chest or abdomen	Apnea, cough, and deep breathing	Piezoelectric sensor	Bluetooth, Mobile app, Fish lateral line structure, PVDF ^7^, Low detection limit 0.5 mN, Sensitivity 0.24 V/N, Response time 4 ms	[37]
Patch on chest	RR	Piezoelectric	PVDF, Matlab R2022b post processing	[38]
Belt around abdomen or thorax	Sleep monitoring	Textile RIP ^8^ sensor	Digital frequency-counting algorithm, Wireless communication, 800 mAh Li-pol battery, 380 kHz resonance frequency, Peak consumption 140 mW	[39]
Chest belt	Respiratory flow, RR, RV	Capacitive sensor, accelerometer	Bluetooth, IMU, Motion correction, Sampling rate 30 Hz	[41]
Chest and abdomen e-textile belts	RR, RV	Capacitive sensor	E-textile sensors, Bluetooth, Estimation error reduction, Operational frequency 100 Hz	[42]
Waist belt	RR	Capacitive sensor	Working pressure range up to 200 kPa, Durability over 6000 cycles	[43]
Chest belt	RR, apnea	Rotating thin-film triboelectric nanogenerator	Retractable self-powered sensor, Wi-Fi, 1 million stretching cycles	[34]
Patch on the chest	Breathing, vital signs, disease progression	Proprietary sensor	Breathing pattern, Tidal volume, HR, BT ^9^, Activity, Wireless communication, Mobile app, AI-Powered disease progression assessment	[20]
Sensor under clothes	Respiration, activity, HR	PPG sensor	Bluetooth, Cellular-based hub, Mobile app	[21]
E-Textile Antenna	RR, HR	RF antenna sensor	Broadband monopole antenna, Conductive fabric	[46]
Textile vest	SCG, BCG, ECG, respiration	Accelerometer, piezoresistive plethysmograph	Accelerometer ST LIS3LV02DL, ±2 g, 12-bit, Textile ECG electrodes, Textile piezoresistive plethysmograph, Sampling rate 200 Hz, Bluetooth	[74]
Body attachment	SCG, ECG, respiration	Accelerometer, piezoelectric respiratory belt	MMA8451Q accelerometer, 14-bit, Sampling rate 800 Hz, Piezoelectric respiratory belt transducer MLT1132, ECG Front-end AD8232, Freescale FRDM-KL25Z acquisition board, FFT processing	[75]
Body attachment	Force- cardiography	Force-sensing resistor	FSR03CE, FCG compared to EDR ^10^ and respiration band, NI-USB6009 DAQ board, 13-bit, Sampling rate 5 kHz	[23]
Clip attached to bra	Respiration, stress, activity	Accelerometer	RR, HR, BLE ^7^	[77]
Chest strap	Respiration monitoring	Accelerometer, Inductive type respiration sensor	Random forest classifier, Sampling rate 1 kHz, 16-bit, Bluetooth, Minimalization of movement artefacts	[79]
Chest mounted	SCG, respiration, apnea	MEMS ^11^ accelerometer	Accelerometer LIS3L02AL, 0–100 Hz, Frequency domain analysis of inspiration, expiration, and apnea.	[82]
Soft skin attached to the neck	Respiratory, swallowing biomechanics	Mechano-acoustic sensors, 2 × IMU	2× IMU units, 200 Hz sampling rate for x,y-axis, 1600 Hz for z-axis, haptic sensor, RR, HR, swallowing, NFC, BLE	[24]
Behind the ear	BCG, PPG, blood pressure	2 capacitive electrodes	BCG—25 mm × 25 mm hybrid sensor for differential sensing and dry electrode for feedback, High-impedance LMC6064 amplifier	[83]
Chest attached	Biopotential sensors, respiration sounds	Biopotential electrodes, piezoelectric microphone	EMG ^12^, Intercostals and diaphragm movement, Microphone: 20–100 Hz, 16-bit, 2.4 GHz wireless communication	[29]
Under the mattress	BCG, HRV and sleep tracking	BCG	RR, HR, HRV, Sleep monitoring, Bed movements, Stress level, Snoring, Sleep quality	[86]
Under the mattress	BCG, HR, RR	Accelerometer, force sensors	Piezoelectric PVDF film and electret polymer material, Sensor location testing	[87]
Mattress	BCG, HR, RR, breathing patterns	Multi-channel optical sensor-array	2× IR LEDs SFH4250 and photodiode BPW34FAS, Dynamic forces modulated the light intensity, NI USB-6009 acquisition unit	[88]
Bed embedded	BCG, pattern recognition, HR, RR	Load cell	Off-the-shelf load cell installed on a typical hospital bed with a ML ^13^ algorithm, Low-cost, Detection rate 83.9%	[91]
Mattress	BCG, HR, RR	Two pressure pads on mattress	BCG evaluation in a sleep monitoring system	[92]
Mattress	BCG, HR, RR, apnea	Set of oil pressure sensors	16-bit, Sampling rate 100 Hz, KSVM ^14^ model, Apnea precision rate 90.46%	[93]
Bed installed	BCG, HR, RR	4 load cells	4× CBCL-6L, Wheatstone bridge, AD8221 amplifier, HR error 2.55%, RR error 2.66%	[94]
Bed	BCG, respiratory disorders	Tensimeters on the bed legs	CNN ^15^ analysis, Accuracy of 96.4%, Sensitivity 92.5%, Specificity 98.1%	[95]
Force plate	BCG, RR, posture	Biomechanical force plate	3D piezoelectric load cells 9286B, Kistler^®^, 600 × 400 mm, Sampling rate 960 Hz, Time warping averaging	[96]
Seat	BCG, HR, RR	Pressure sensor	Air cushion connected to Treston DMP 331	[97]
3 electrodes on the chest	RR, detection of tachypnoea	Impedance pneumo-graph	Dual vector approach	[98]
Electrodes on the chest	RR	EIP ^16^, 3D accelerometer	Adaptive noise cancellation, Band-pass filtering	[99]
Electrodes on the chest	RR, RV	EIP	Segregated envelope and carrier detection	[100]
Integrated circuit	ECG, EEG, RR	EIP ADS129xR	8-channels, 24-bit Analog Front-End, Sampling rate 250 Hz–32 kHz, −115 dB CMRR, Internal oscillator	[101]
Integrated circuit	ECG, respiration	EIP AFE4960	2 channels, 22-bit, Single ADC, SPI and I^2^C interface, Sine wave or square wave excitation	[102]
Integrated circuit	ECG, optical HR, respiration	EIP AFE4500	4 input channels, 22-bit, single ADC, SPI and I^2^C interface	[103]
Integrated circuit	ECG, respiration, pace detection	EIP ADAS1000	5 acquisition channels and one driven lead, Serial interface SPI/QSPI, AC and DC lead-off detection	[104]
Integrated circuit	ECG, respiration, pace detection	EIP MAX30001	High Input Impedance (>1 GΩ), High-Speed SPI interface, 32-Word ECG and 8-Word BioZ FIFOs, EMI filtering, ESD protection, DC leads-off detection	[105]
Integrated circuit	PPG, ECG, BioZ, EDA	EIP AS7058	2 ADC (20-bit) for PPG acquisition, 1 ADC (20-bit) for ECG/BIOZ acquisition, SPI and I^2^C interface	[106]
Chest belt	HR, RR, BP, PWT	400 µm multimode OF ^17^	Laboratory testing, HRV 2.5%, NA ^18^ = 0.5, Single digital camera for signal acquisition	[118]
Chest belt	RR	D-shaped POF ^19^	RR under different movement	[126]
Chest belt	RR	POF sensor	Error 3 min^−1^	[125]
Chest belt	RR	FBG ^20^ sensor	Tested wavelengths 525, 660, 850, 1310, 1550 nm MRI ^21^ compatible, Elongation up to 3%	[22]
Textile	RR, apnea	Two FBGs	RR during sport, 10 mm of grating length,	[116]
T-shirt	HR, RR	Three FBGs glued on the textile with silicone rubber	Highly stretchable and compressible	[107]
Mattress	HR, RR, activity	POF sensor	HR error 2 min^−1^, RR error 1 min^−1^	[127]
Mattress embedded	RR	4 × 4 matrix structures of POFs	645 nm and silicon photodiode, Arduino Resolution 2.2–4.5%/N	[128]
Smart bed	ECG, HR, BP ^22^, PPG, BT	Inspired O_2_ FBG in fabric	Monitoring patient under MRI	[117]
Chest belt	RR	POF-GPL ^23^ sensor	Polymethylmethacrylate core with a diameter of 485 μm, Base material-thermoplastic polyurethane	[129]
Chest belt	RR HR	Multimode silica OF with an elastomer OF	Filtering 0.1 Hz to 0.4 Hz	[121]
Smartphone	RR	Smartphone-integrated POF	Cloud connectivity	[142]
Chair back	BCG, HR, RR	Microbend OF	Gaussian mixture model and classification based on K-Nearest Neighbors, Accuracy 94.6%.	[122]
Radar	HR, RR, athletes monitoring	Stationary parabolic antenna	Operational frequency 24.1 GHz, Transmitter output 30 mW, Radius 0.6 m, Antenna gain 40 dB	[130]
Micro-radar	RR	Wearable neck pendent radar	Operational frequency 24 GHz, Wi-Fi communication	[131]
Radar	SCG, HR, suitable for RR	Two stationary antennas	Operational frequency 5.8 GHz, Transmitting power 6 dBm	[132]
Radar	BCG, RR, HR	Stationary antenna	Operational frequency 24 GHz, Transmitter power output 35 mW	[133]
Antenna worn on the chest or abdomen	RR, RV, HR	Monopole helical antenna	Operational frequency 1.82/1.90 GHz, Transmitting power 12.84/10.42 dBm	[134]
Camera system	RR, HR, HRV	Commercial camera	Motion compensation, Two-phase temporal filtering, Signal pruning	[135]
Camera system	RR, RV	Infrared cameras	Twelve retro-reflexive markers, 8 IR cameras, Sampling rate 100 Hz	[136]
Camera system	RR	Infrared camera	Tracking region of interest, Mean shift localization	[137]
Camera system	RR, HR	Infrared camera	Long-wave IR sensing, Wavelet analysis, Thermal sensitivity of 0.025 °C, 14-bit dynamic range	[138]
Camera system	RR	Infrared camera	Thermal sensitivity of 0.08 K, 50 fps	[14]
Camera system	Respiration phases	Infrared camera	FLIR A325sc with 50 μm lens, 60 fps, Resolution 320 × 240, No image segmentation	[140]

^1^ Bluetooth low energy, ^2^ Inertial measurement unit, ^3^ Respiration rate, ^4^ Heart rate, ^5^ Heart rate variability, ^6^ Respiration volume, ^7^ Polyvinylidene fluoride, ^8^ Respiratory inductance plethysmography, ^9^ Body temperature, ^10^ ECG-derived respiratory, ^11^ Microelectromechanical systems, ^12^ Electromyography, ^13^ Machine learning, ^14^ Kernel Support Vector Machine, ^15^ Convolutional neural network, ^16^ Electro-impedance plethysmography, ^17^ Optical fiber, ^18^ Numeric aperture, ^19^ Plastic optical fiber, ^20^ Fiber Bragg Grating, ^21^ Magnetic resonance imaging, ^22^ Blood pressure, ^23^ Grooved, photosensitive, luminescent.

## 3. ECG-Derived Respiration

ECG-derived respiration (EDR) (Table 2) serves as a non-invasive and cost-effective method for estimating the respiratory rate from an ECG signal. This estimation can be conducted in either the time or frequency domain [7,143,144]. As an individual breathes, the electrodes measuring the ECG undergo dynamic movements in distance and direction, mirroring breathing in and out [145]. These movements induce variations in the QRS of the ECG signal, allowing for the estimation of RR. In the time domain, ECG amplitude and HRV are combined, employing cubic spline interpolation and amplitude detection to enhance estimation accuracy [146,147]. The frequency-domain approach involves using a band-pass filter to capture the ECG spectrum around 0.3 Hz (covering the respiratory frequency), with the respiratory rate determined by identifying the spectrum peak. This peak corresponds well to the respiratory signal spectrum.

In contrast to other invasive and costly methods, ECG-derived respiration estimation provides a more comfortable and cost-effective approach to measuring respiratory rate. Nevertheless, current methods for ECG-derived respiration estimation often encounter challenges related to low accuracy or high computational complexity.

### 3.1. Determination of EDR from Amplitude

Different approaches have been proposed in the past to obtain RR from ECG and one of them is the derivation from ECG amplitude. This method is highly preferred, for example, in ICU patients, where the RR can be simultaneously monitored and derived using the maximum amplitude variation (PAV) from the single-lead ECG signal [146].

Overall, to achieve a capable breathing result, the signal needs to be filtered, as it is often corrupted by low-frequency artifacts from ECG fluctuations or high-frequency noise and interference from power lines. Several algorithms have also been developed to estimate respiration from ECG [148,149,150,151]. Lenis et al. [151] proposed a new algorithm for an optimal linear combination of different EDR methods. This would lead to an even more accurate result, which cannot be achieved by a separate method. Several algorithms have also been developed to estimate respiration from ECG and 13 methods have been implemented—six deal with the amplitude modulation caused on the QRS complex (first principal component (PCA), QRS integral, QR slope, QRS width, R peak, and RS amplitude), four focus on the T wave (T integral, T peak, T slope, and peak RT), another uses the statistical properties of the ECG in each beat, another extracts the spectral properties of the signal below 0.5 Hz using a discrete wavelet transform, and the last one uses the time interval between individual QRS complexes (RR interval). The top four methods for EDR were PCA, R peak, QRS integral, and RS amplitude, all based on the QRS complex. Widjaja et al. [152] presented an improved algorithm based on kernel principal component analysis (kPCA). They compared their results with a reference respiratory signal using correlation and coherence coefficients and found that kPCA outperformed other methods and was suitable for accurate sleep apnea detection and home monitoring. Also, Langley et al. [153] presented the PCA algorithm as a suitable method for deriving RR from ECG amplitudes. In other studies, Varon et al. [154] focused on the development of ambulatory systems capable of cardiorespiratory monitoring. They compared 10 EDR methods for calculating respiration from single-lead ECG. They found that methods based on QRS slopes are the most suitable.

Alam et al. [155], for example, in their work pointed to the possibility of deriving respiration from an ECG wrist wearable, more specifically respiration rate (RR) and minute ventilation (VE) parameters. Such measurement is easy for the wearer in various situations, is comfortable, and does not interfere with long-term monitoring. They collected data during physical activities to show the ability of the device to measure in real life and obtained very satisfactory results. Another study describes the respiratory frequency derived from the ECG in a group of survivors of acute myocardial infarction. They found that this method is suitable and accurate in comparison with the direct measurement of respiratory frequency. They also developed an algorithm to calculate the mean respiratory rate from 10 min ECG recordings [156]. They used not only QRS complex amplitude but also QRS vectors and RR intervals to derive respiration from the ECG. Lazaro et al. [157] presented a wearable wristband capable of deriving RR as well as tidal volume (TV). EDRs were studied from the morphology of the QRS complex: QRS slope range (SR), R-wave angle (Π), and R-S amplitude (RS). The device is suitable for continuous monitoring. Klum et al. [158] measured a chest ECG sensor and followed the placement of electrodes with high signal correlation with gold standards. To calculate RR in each position, they implemented three EDR algorithms derived from HRV and QRS amplitude and an algorithm based on linear PCA. The linear PCA method outperformed the other two methods, followed by the QRS amplitude concept. They showed that it is possible to obtain RR even from various unfavorable positions and which positions it is worthwhile to measure the ECG to obtain the best EDR signals. Basically, they confirmed what we also found in our earlier publication about the placement of ECG electrodes. We mentioned methods for deriving the respiratory signal from the ECG using morphological variations between individual beats based on QRS complex amplitude, R wave duration, QRS complex area, T-wave amplitude, or T-wave area. ECG-derived breathing is principally derived from chest movements and changes in the impedance distribution of the human chest, which affects the amplitude of the QRS complex, which we also found in our research [27].

### 3.2. Determination of EDR from HRV

HRV is the result of various influences of the autonomic nervous system on heart rate. The impact of respiration rate on HRV, known as respiratory sinus arrhythmia (RSA), is widely acknowledged. However, the specific effects of different respiration rates on HRV are not fully understood [159,160,161,162,163]. RSA is characterized by HRV synchronized with respiration, where the RR interval is shortened during inspiration and lengthened during expiration [164]. RSA falls under the category of cardiorespiratory interactions, which are classified into three types: RSA, defined by heart rate variability at the breathing frequency; cardio-ventilatory coupling, characterized by synchronization between the heartbeat and the onset of inspiration; and respiratory stroke volume synchronization, characterized by a constant phase difference between the right and left stroke volumes over a respiratory cycle [67,165]. The most common wearable electronics sensor measuring RSA is the PPG sensor. Good and statistically relevant research was conducted by Natarajan et al. [166] who measured RR using the wearable device Fitbit Charge from the power spectral density of HR from sleep studies. They achieved RMS error = 0.648 min^−1^. The standard used in most wearable devices has become the Whoop algorithm [167]. A very good study was performed by Berryhill et al. [168].

A certain problem of this algorithm and the RSA process, in general, is that it only works reliably in a state of rest. It perfectly determines RR during sleep, but during physical activity or even talking, the diagnosis of RR is problematic. That is why researchers are constantly working on improving the methodology. Among the relevant research, we can mention, for example, the work of Karlen et al. [169], who computed the RR using the Incremental-Merge Segmentation algorithm and FFT and achieved an RMS error of 3 ± 4.7 min^−1^ and Schäfer and Kratky [147] who compared different techniques and achieved the best error of 0.84 min^−1^. Bian et al. [170] used deep learning and obtained a mean absolute error of 2.5 ± 0.6 min^−1^. Dubey et al. [171] used a Spectral kurtosis-based method with an RMS error of 1.2 ± 0.3 min^−1^. Dai et al. [172] described an algorithm based on CNN to estimate the RR in the presence of motion. Shuzan et al. [173] used machine learning with a mean absolute error of 1.91 min^−1^. A novel and robust technique based on a fusion algorithm, which improves existing methods, with probabilistic estimation for clinical practice was presented by Pimentel et. al [174]. In [175], they determined the RR from PPG using infrared and green colors. During this process, they considered 12 potential parameters, including pulse width variability, pulse amplitude, and a data fusion model that utilizes five different PPG features to obtain real-time RR. Testing was conducted in various body positions, specifically on the arm, wrist, and ankles. Suleman et al. [176] devoted themselves to respiratory event estimation from PPG using a simple peak detection algorithm. An important contribution to the deployment of machine learning in this issue was written by Beh et al. [177].

With the advancement of miniaturization and the decrease in electricity consumption, ring-shaped PPG sensors are gaining momentum. As one of the first and leading companies, we can consider Ouraring (Oura Health, Oulu, Finland), which also calculates RR from RSA [178]. The upcoming Galaxy ring (Samsung Electronics Co., Yeongtong-gu Suwon, Gyeonggi, Republic of Korea) sounds very interesting [179].

In the future, we can count on the development of processors specially designed for ECG-derived respiration. The study by Fan et al. [9] introduces an innovative processor specifically designed for achieving high accuracy and ultra-low power in EDR estimation. Various techniques, including QRS detection with refractory period refreshing and adaptive threshold EDR estimation, have been proposed to enhance accuracy and reduce computational complexity, subsequently lowering power consumption. Fabricated using 55 nm processing technology, the proposed processor demonstrates a remarkable EDR estimation error of 0.73 on the CEBS database and 1.2 on the MIT-BIH Polysomnographic Database. Notably, it achieves a record-low power consumption of 354 nW for respiration monitoring. This superior performance positions the proposed processor as an ideal candidate for integration into wearable sensors, facilitating ultra-low power and highly accurate respiration monitoring.

**Table 2:** ECG-derived respiratory monitoring.

Sensor Type	Application	Sensing Element	Key Parameters	Ref.
Wrist-worn EDR ^1^	RR, ventilation	ECG ^2^, IMU ^3^ sensors	For asthma patients, IMU sample rate 250 Hz, Using during physical activity	[155]
Armband EDR	RR ^4^, tidal volume	ECG	EDRs from the morphology of the QRS complex: QRS slope range, R-wave angle, R-S amplitude	[157]
Chest sensor	RR, HRV ^5^	ECG electrodes	3 EDR algorithms from ECG	[158]
Wrist wearable EDR	RR, HRV, sleep studies	PPG ^6^	Fitbit Charge, Power spectral density of HR, RMS ^7^ error = 0.648 min^−1^	[166]
Mobile phone camera EDR	RR, HRV	Mobile phone camera	Incremental-Merge Segmentation algorithm, FFT ^8^, RMS error 3 ± 4.7 min^−1^	[169]
EDR from ECG ^6^	RR, HRV	ECG	Compared different techniques, Best error of 0.84 min^−1^	[147]
EDR	RR, HRV	PPG dataset	Deep learning, MAE ^9^ 2.5 ± 0.6 min^−1^	[170]
Wrist wearable EDR	RR, HRV	PPG	556 nm LED ^10^, Spectral kurtosis-based method, RMS error 1.2 ± 0.3 min^−1^, BLE ^11^	[171]
Wrist wearable EDR	RR, HRV	PPG	CNN ^12^ algorithm, RR in the presence of high activity	[172]
Wrist wearable EDR	RR, HRV	PPG dataset	Different Machine learning, Sampling rate 500 Hz, MAE 1.91 min^−1^	[173]
EDR	RR, HRV	PPG, ECG, accelerometer	Fusion algorithm, Probabilistic estimation for clinical practice	[174]
Arm, wrist, ankles EDR	RR, HRV	PPG	IR/green LEDs, 12 parameters, Data fusion model of 5 PPG features, Various postures	[175]
EDR	RR	Capnobase and PPG dataset	FFT analysis and peak detection, MAE 2.14 ± 5.59 min^−1^ and 1.59 ± 3.21 min^−1^	[176]
Ring EDR	RR, HRV, sleep, BT ^13^, activity	PPG	Oura ring, BLE	[178]
Ring EDR	RR, HR, ECG, activity, BT	PPG	Galaxy ring, BLE, NFC ^14^	[179]
Processor for wearable sensors	RR, HRV	EDR estimation	QRS detection with refractory period refreshing, Adaptive threshold, 55 nm technology, Estimation error 0.73, Power consumption 354 nW	[9]

^1^ ECG-derived respiration, ^2^ Electrocardiography, ^3^ Inertial measurement unit, ^4^ Respiration rate, ^5^ Heart rate variability, ^6^ Photoplethysmography, ^7^ Root mean square, ^8^ Fast Fourier transformation, ^9^ Mean absolute error, ^10^ Light emitting diode, ^11^ Bluetooth low energy, ^12^ Convolutional neural networks, ^13^ Body temperature, ^14^ Near field communication.

ECG-derived respiration sensors are particularly suitable for continuous monitoring of respiration patterns and provide valuable information about respiration across different activities and time periods. They are almost exclusively intended for the calculation of RR. RV can be partially determined only by monitoring amplitude changes. They can often be found as part of smartwatches and rings. The advantage is that they are easily available to the general public for daily monitoring of basic physiological functions, such as breathing as well as monitoring heart rate or saturation. The multifunctionality brings the wearer a comprehensive picture of their current physiological state and health. These sensors have the disadvantage of the difficulty of extracting accurate information from the signals precisely because of their complex nature, and advanced signal-processing techniques are required to distinguish other physiological signals. ECG-derived respiration sensors typically operate at moderate sampling rates and moderate power efficiency.

## 4. Acoustic-Based Methods

Respiratory sounds arise due to turbulent air flow in the larger airways [180,181]. They stem from pulmonary vibrations transmitted to the thoracic wall through the corresponding airways. The nature of sounds during normal breathing varies based on the acquisition location and the phase of the ventilatory cycle [15]. Respiratory sounds, resulting from air movement within the respiratory system, serve as an initial means of detecting respiratory illnesses. Traditionally, these sounds are identified by trained physicians using a stethoscope, and their frequency typically ranges from 20 to 1000 Hz [182]. Auscultation of respiratory sounds is typically conducted near the airways or throat using stethoscopes or microphones. However, there have been attempts to record respiratory sounds from greater distances using precise external microphones or simpler methods using mobile phones (Table 3).

Additionally, noise-reduction devices, pre-processing tools, and audio-cleaning devices are available to facilitate sound acquisition for both patients and physicians. Automation of this process could lead to the development of electronic tools supporting the healthcare system. A great overview of acoustic wearable respiration sensors can be found in the reviews by Daiana da Costa et al. [15] and Trocoso et al. [5]. As for the approach to acoustic signal processing, in addition to normal filtering, spectral analysis, and the extraction of various parameters, neural networks are also an integral part. Excellent reviews of the processing of respiratory sounds were performed by Kim et al. [183] and Acharya et al. [184].

### 4.1. Electronic Stethoscopes

Certain research groups leverage electronic stethoscopes to generate input data for subsequent analysis or classification utilizing machine-learning algorithms, such as CNN or SVM. Their objective is to identify and contribute to the diagnosis of respiratory disorders [185,186,187]. Various commercial electronic stethoscopes are available, with the Mintti Smartho-D2 [28] being an illustrative example used in studies by Liu et al. [188] and Emmanouilidou et al. [189]. These studies evaluated children’s respiration in challenging environments. Some researchers have even developed their own electronic stethoscopes. For instance, Aykanat et al. [190] constructed their electronic stethoscope using a compact and directional microphone. The copious amount of recorded data undergoes a feature-extraction process employing machine-learning techniques. Perhaps the most advanced digital stethoscope was presented by Lee et al. [191]. They presented a soft wearable system that facilitates real-time, wireless, and continuous auscultation. This innovative system serves as a quantitative diagnostic tool for various diseases. The soft device is capable of detecting continuous cardiopulmonary sounds with minimal interference and classifying real-time signal abnormalities. Through a clinical study involving multiple patients and control subjects, they highlight the unique advantages of the wearable auscultation method, leveraging embedded machine learning for automated diagnoses of four lung diseases: crackle, wheeze, stridor, and rhonchi, achieving an impressive 95% accuracy. The system also shows potential for home sleep studies. The AI-Ready infrasound Stethoscope VoqX (Sanolla, Nesher, Israel) for the evaluation of sounds in the range of 3–40 Hz is presented by Sanolla [192]. This device is also enhanced by PPG and BT sensors. Emokpae et al. [193] present the WearME system, which uses a body area sensor network on the chest, with each node having a suite of sensors including a digital stethoscope, an ECG monitor, a temperature sensor, and a body posture tracker.

### 4.2. On-Body Microphones

Wearable systems for acquiring respiratory sounds commonly employ wireless acoustic sensors worn by patients, connected to smartphones for data storage. Mostly, the evaluation of respiratory parameters is performed directly on the mobile phone. However, there are also variants where signal analysis takes place directly in the embedded electronics of the microphone device. Fang et al. [194] presented a respiratory sound-measurement system designed to detect sleep apnea in infants in the nose area. Very similar research was performed by Werthammer et al. [195], but they focused on infants and compared the result with transthoracic impedance and ECG monitoring. On the other hand, Oletic et al. [196,197,198] monitored asthma while considering energy efficiency. Very extensive research in this area was performed by Reyes et al. who, for example, detected crackle [199] and tracheal sounds [200]. Corbishley and Rodriguez-Villegas [201] proposed a miniaturized and wearable respiration-monitoring system utilizing a neck-mounted microphone with an aluminum conical bell to capture respiratory acoustics effectively. In the current version, they still analyzed the signal in PC. Great potential has been shown by microphones made of flexible materials such as the respiration sensor using flexible piezoelectric film on a soft compliant substrate from Liao et al. [202] or research by Yilmaz et al. [203], who used a contact microphone from a piezoelectric film in silicone rubber to detect diaphragm movement and thoracic sounds. An advanced piezo microphone design was also presented by Chen et al. [204]. The development of BodyScope aimed to capture sounds from the throat region and categorize them into specific activities such as eating, drinking, speaking, laughing, and coughing [205]. The device, created by modifying a wireless headset with a microphone and a stethoscope chest piece, minimizes external audio interference. The sensor is strategically placed close to the carotid artery region, as indicated by preliminary test results, and transmits audio signals to a computer or smartphone. Li et al. [206] introduced a real-time wheeze detector, comprising a wireless sound acquisition module, a wearable mechanical design, and a host system. The sensor module included an omnidirectional condenser microphone and a stethoscope bell. Their main goal was to achieve low computational complexity. In the publication, one can also find a table with competing products. In [17], a wireless microphone was utilized as a portable, cost-effective, and user-friendly wearable device positioned adjacent to the nose. The objective was to measure respiratory rates during sleep. The microphone, affixed near the nose using tape, transmitted signals wirelessly to a smartphone. Certain devices not only record respiratory sounds but also capture chest movements [207,208,209]. Among the commercially available alternatives, we can mention, for example, products Respa (Zansors, LLC, Arlington, VA, USA) [210], which can be simply clipped onto clothing while using a microphone to pick up sounds from the nose and mouth. The device is equipped with a 3D accelerometer, magnetometer, and barometer. A very simple standalone device for identifying RR in the wild was introduced by Taylor. et al. [211]. Another nice product is the Sylvee wearable patch (Respira Labs, Mountain View, CA, USA) [212] which was conceived for evaluating individuals with COPD, COVID-19, and asthma using integrated speakers and microphones. These components gauge alterations in acoustic resonance on the lower part of the rib cage.

Elfaramawy et al. [213] designed a wearable patch sensor network for evaluating RR and couching events. The system uses a low-power inertial measurement unit (IMU) to quantify the respiratory movement and a MEMs microphone to record respiratory sounds. Various research groups have developed multimodal wearable systems capable of recording breath sounds along with heart rate, ECG, or oxygen saturation [214,215]. Yin et al. [216] employed an IoT system for monitoring athletes’ RR based on speech recognition technology. Respiratory motion will affect the changes in the ECG. They combined HR with respiratory sounds. Health Care Originals presented a device called ADAMM (Health Care Originals, Rochester, NY, USA) [217], which is a cough-, wheezing-, RR-, HR-, BT-, and movement-identifying smart patch. As for the number of microphones, it is likely no one can compete with Fraunhofer IKTS [218], which created a textile vest integrating a matrix of piezoceramic acoustic sensors in the front and back of the thorax. They also designed their own machine-learning algorithms to analyze such complex signals.

Attention is also paid to the issue of acoustic sensor–skin contact. Cotur et al. [219] propose a stretchable wearable consisting of a silicone membrane housing a microelectronic sensor. Acoustic waves are transmitted through water or hydrogel inside the silicone capsule. This sensor exhibits promise in heart monitoring, with preliminary findings in respiratory sound recording. Chen et al. [220] crafted an e-skin-based wearable with monitoring and sound alarm functions, detecting physiological signals for cardiovascular diseases or sleep apnea monitoring. Ni et al. [221] presented an innovative automated multiparametric respiratory and vital-sign-monitoring system for clinical and home environments. The technology employs soft, skin-mounted electronic miniaturized motion sensors. This setup facilitates precise, wireless measurements of mechanoacoustic signatures associated with core vital signs (HR, RR, and BT) as well as less-explored biomarkers (such as coughing count), ensuring accuracy and resilience to ambient noises.

### 4.3. Remote Microphones

All research so far has been interesting and had the common denominator of trying to capture respiratory sounds as high as possible. This brings with it a small disadvantage, which is that the acoustic sensors must be attached to the body or worn very close to the respiratory tract. But there is also a remote approach where they detect respiration acoustically from a greater distance. Of course, the input sound quality will be lower and will fluctuate with the changing distance, but on the other hand, such a system will not burden the examined person at all. Most research logically focuses on mobile phones. Most of us carry mobile phones every day, and the quality of microphones and the computing power of mobile phones are growing exponentially. Among the typical approaches, we can mention, for example, Markandeya et al. [222] who monitored sleep apnea and Nakano et al. [223] who also monitored sleep apnea but focused on studying snoring as a crucial sound indicative of these conditions. Barata et al. [224] monitored asthmatic coughs and cough epochs. Bokov et al. [225] monitored respiratory sounds, especially wheezing, in pediatric patients and Nam et al. [226] estimated RR using a headset. Xue et al. [227] tested different algorithms for classifying respiratory sounds measured from a distance. They first worked with databases, but later they also tested system based on a Renesas synergy internet-of-things (IoT) platform in a hospital.

**Table 3:** Acoustic-based respiratory monitoring.

Sensor Type	Application	Sensing Element	Key Parameters	Ref.
Stand-alone stethoscope	Respiratory sound analysis	Digital stethoscope	10 Hz–2 kHz, 100× amplification, Bluetooth for mobile phone connection, DSP ^1^, AI ^2^, Ambient noise cancelation, Real-time audio curve display, 150 g, 3.5 mm jack	[28]
Stethoscope connected to PC	Spectrogram classification	Own directional microphone	Directional microphone, Lubricated contact area, SVM ^3^ and CNN ^4^ algorithm, 3.5 mm jack	[190]
Wearable stethoscope	Advanced sound signal analysis	Soft and flexible wearable smart patch system	36 Hz–2 kHz, SNR ^5^ 14.8 dB, Real time abnormalities, 95% accuracy, Controlled motion artifact, BLE ^6^	[191]
Stand-alone Stethoscope (Classic design)	Recording and data transmission	Digital stethoscope	3–40 Hz, 40× amplification, Ambient noise cancelation, Sound signature spectrogram, Integrated HR, SpO_2_, BT and respiratory cycle, 48 h work time, Accuracy 92%	[192]
Body area network of stethoscopes	Advanced signal analysis	Body sensor area network	Strap or shirt option, SNR 48 dB, Integrated ECG monitor, BT, and body posture tracker	[193]
Body worn connected wireless to mobile phone	Asthmatic wheeze quantification	Digital MEMS microphone	ADMP441, I^2^C, Sensitivity −26 dBFS, Power consumption: 216–357 μW (signal streaming), 320–420 μW (classification on sensor), SNR 50–62 dB, Power 2520 μW at 1.8 V, Bluetooth	[198]
Body worn connected wireless to mobile phone	Asthmatic wheeze quantification	Electret condenser microphone	KEEG1542, Sensitivity −42 dB, Power consumption: 216–357 μW (signal streaming), 320–420 μW (classification on sensor), SNR 50–62 dB, Power 1000 μW @ 2.0 V, Bluetooth	[198]
Body worn connected wireless to mobile phone	Asthmatic wheeze quantification	Analog accelerometer	ADXL337, Sensitivity 300 mV/g, Power consumption: 216–357 μW (signal streaming), 320–420 μW (classification on sensor), SNR 50–62 dB, Power 900 μW @ 3.0 V, Bluetooth	[198]
Body worn connected wireless to mobile phone	Asthmatic wheeze quantification	Analog MEMS microphone	ADMP404, Sensitivity −38 dBV, Power consumption: 216–357 μW (signal streaming), 320–420 μW (classification on sensor), SNR 50–62 dB, Power 375 μW @ 1.5 V, Bluetooth	[198]
Body worn connected wireless to mobile phone	Asthmatic wheeze quantification	Digital accelerometer	ADXL345, SPI, Sensitivity 3.9 mg/LSB, Power consumption: 216–357 μW (signal streaming), 320–420 μW (classification on sensor), SNR 50–62 dB, Power 350 μW @ 2.5 V, Bluetooth	[198]
Body worn connected wireless to mobile phone	Asthmatic wheeze quantification	Analog MEMS microphone	ICS-40310, Sensitivity −37 dBV, Power consumption: 216–357 μW (signal streaming), 320-420 μW (classification on sensor), SNR 50–62 dB, Power 16 μW @ 1.0 V, Bluetooth	[198]
Body worn connected wireless to mobile phone	Asthma monitoring	Audio amplifier	MSP430 microcontroller, SPP, Orthogonal Matching Pursuit algorithm, Accuracy 80%, Bluetooth, 8 kb/s streaming	[195]
Body worn connected wireless to mobile phone	Asthmatic wheeze detection	Microphone or accelerometer	TMS320C5505, DSP, Accuracy 92%	[196]
Body worn connected to mobile phone using audio cable	Crackle sound detection	Electret microphone in plastic bell capsule	Microphone BT-2159000, Accuracy 84.68–89.16%	[199]
Body worn connected to mobile phone using audio cable	Tracheal sounds acquisition	Electret microphone	Microphone BT-21759000, 50–3000 Hz Correlation index for RR r^2^ = 0.97,	[200]
Neck-mounted connected to PC using audio cable	Breathing sounds	Microphone with aluminum conical bell	Microphone MD4530ASZ-1, 100–5000 Hz, Sensitivity −42 dB, Breathing detection accuracy 91.3%	[201]
Six wearable stethoscopes in vest	Diaphragm movement, sounds detection	Piezoelectric film in silicone rubber	ADC converter AD7988, Sampling rate 5 kHz, SPI	[203]
Chest worn microphone connected to PC using cable	Lung and heart sounds	Piezoelectric microphone	Ultrasensitive accelerometer, 9.2 V/g, 20–1000 Hz, LMP7721 amplifier, SNR 42–59 dB	[204]
Chest worn connected to PC or mobile phone	Activity recognition	Microphone	Activity identification accuracy 71.5%	[205]
Chest worn wireless connected to PC	Wheeze detector	Condenser microphone in stethoscope bell	TS-6022A, 500× amplification, 12-bit ADC–MSP430 processor, sampling rate 2 kHz, Bluetooth	[206]
Microphone fixed near nose connected wireless to mobile phone	Sleep RR detection, OSA ^7^	Microphone	RR detecting accuracy 98.4%, OSA detecting accuracy 97.44%	[17]
Chest worn nanosensor	Mechano-acoustic cardiopulmonary signals	High-precision vibration sensor	Hermetically-sealed high-precision vibration sensor, Nano-gap transducers, 2 × 2 mm microsensor, 0.5 Hz–12 kHz, 10 μg–16 g, Sensitivity 76 mV/g	[208]
Clipped onto clothing wireless connected to mobile phone	Sound from nose/mouth, breathing	Microphone	Microphone, 3D accelerometer, Magnetometer, Barometer, Commercial, Bluetooth	[210]
Contact microphone on chest strap	HR, RR	Piezoelectric microphone	20–200 Hz, L496ZG microcontroller, Power consumption 14.85 mW, HR Median percentage error 0.33%	[211]
Wireless thoracic and abdominal patch sensors with wireless communication to PC	Cough detection and RR	IMU and MEMS microphones	LSM9DSO IMU, ADMP401 MEMS microphone, SNR 62 dBA, MSP430 microcontroller, Power consumption 40–53.5 mW	[213]
Multiparameter cardiopulmonary acquisition device worn on shoulder	Breathing sound	Microphone in stethoscope bell	JL-0627C microphone, 12-bit, Bluetooth, Accuracy for RR 96.5%, Integrated ECG, SpO_2_ under motion 6-h working time	[213]
Multimodal chest sensors in vest	Bioimpedance tomography, RR, chest sounds	Electret microphones and chest impedance sensors	Integrated ECG, SpO_2_, Accelerometer, Bluetooth, 6-h working time,	[214]
Flexible wireless patch on upper torso	Detection of cough, RR, wheeze	Microphone and accelerometer	Integrated HR, BT, and activity level, Bluetooth	[217]
Textile pneumo vest with acoustic sensors	Lung function monitoring	Matrix of piezoceramic sensors	ML ^8^ algorithm	[218]
Soft skin-chest mounted wireless sensor	HR, RR, BT, and cough detection	Miniaturized mechanoacoustic motion sensors	LSMDSL IMU ^9^ sensor, Elastomer membrane, BLE, Immune to ambient noise, CNN network	[221]
Sound detection from distance	OSA	Mobil phone	iPhone 7 calibrated by oesophageal pressure manometry, ML algorithm, Prediction of ΔPes ^10^ with MAE ^11^ 6.75 cm H_2_O, r = 0.83	[222]
Mobile phone on the chest	OSA, snoring	Mobil phone	FFT ^12^ analysis, Online analysis on mobile phone, Snoring time correlation r = 0.93, Apnea-hypopnea index correlation r = 0.94, OSA sensitivity 0.7, OSA specificity 0.94	[223]
Sound detection from distance	Asthmatic coughs and cough epochs	Mobil phone	CNN model, Gaussian mixture models, Matthew’s correlation coefficient 92%, Cough epochs count difference 0.24	[224]
Mobile phone near mouth	Pediatric wheezing	Mobile phone	SVM algorithm, Sensitivity 71.4%, Specificity 88.9%	[225]
Mobile phone on the neck	RR	Mobile phone or headset	iPhone 4s–30 cm away from nose, PSD ^13^ calculation, Median error < 1%	[226]
IoT device in distance	Cough, breath, and wheeze analysis	Microphone	Embedded system, Renesas S5D9 120 MHz, Kernel-like minimum distance classifier, Accuracy up to 91.23%	[227]

^1^ Digital signal processing, ^2^ Artificial intelligences, ^3^ Support vector machine, ^4^ Convolutional neural network, ^5^ Signal to noise ratio, ^6^ Bluetooth low energy, ^7^ Obstructive sleep apnea, ^8^ Machine learning, ^9^ Inertial measurement unit, ^10^ Peak to through differences, ^11^ Median of absolute error, ^12^ Fast Fourier transformation, ^13^ Power signal density.

Wearable acoustic sensors for breathing monitoring include microphones and digital stethoscopes built into wearable devices or even mobile phones. It is a unique approach that, in addition to standard RR detection, also monitors breathing diseases such as asthma, sleep apnea, or coughing. These sensors manage to detect even very subtle changes in breathing as they are very sensitive, although a higher frequency may be needed to record detailed breathing patterns. This makes them very valuable for remote, round-the-clock patient monitoring and telemedicine applications in general. Because they require minimal contact with the body and are versatile in terms of incorporation into various devices, they are comfortable to use. Their disadvantage is interference with ambient noise, so it is important to filter out these unwanted sounds, which is quite complex and requires the use of advanced algorithms for signal processing. Depending on the application, these sensors can work at different sampling rates.

## 5. Parameters of Exhaled and Blood Gases

The composition of exhaled and blood gases offers valuable insights into respiratory efficiency and overall physiological health. Examining both exhaled and blood gases provides a more comprehensive picture of an individual’s respiratory and metabolic status and guides appropriate interventions and treatment strategies (Table 4).

Airflow-based sensors operate through direct interaction with the airflow to assess respiratory rate (RR), respiratory volume (RV), and other spirometry parameters. These sensors are also used to determine breath composition. They are strategically positioned near the upper airways, often integrated into respiratory facemasks, nasal cannulas, or headsets. Integrating airflow-based sensors into wearable devices enables the continuous collection of breath data over extended periods. Thermal imaging cameras can also be utilized to capture temperature variations during respiration without direct contact.

In the ever-evolving healthcare and technology environment, innovative technologies open up new possibilities for personalized healthcare and provide a basis for more effective strategies in combating respiratory challenges, with a particular focus on respiratory diseases like COVID-19 [228]. Viral agents stimulate the production of volatile organic compounds (VOCs), which are detectable in exhaled breath. As a result, these compounds act as swift diagnostic biomarkers for COVID-19.

Understanding the blood gas parameters of an individual is a crucial tool in critical care, aiding in diagnosing and managing respiratory distress, acidosis, and alkalosis. Pulse oximetry is employed for monitoring blood oxygen saturation and assessing oxygen partial pressure (PaO_2_) without the need for invasive arterial blood gas measurements. A popular non-invasive method used for measuring the partial pressure of oxygen and carbon dioxide in the blood involves transcutaneous blood gas monitoring [229].

### 5.1. Composition of Breath Gases

This section of the article focuses on breath gas analysis. Breath analysis has gained attention as a modern diagnostic method for the early detection of physiological changes. This method is non-invasive and painless with the possibility of long-term observation [230]. Most of the exhaled breath volume is made up of nitrogen (78%), oxygen (16%), carbon dioxide (4%) and water. Volatile organic compounds (VOCs) are also present in exhaled air, although their concentration is minimal [231,232]. However, these VOCs can provide valuable insights into physiological and pathological function, serving as biomarkers for the non-invasive recognition of numerous diseases. The main biomarkers that provide the identification of human diseases include ammonia, toluene, pentane, acetone, isoprene, nitric oxide, and other compounds such as methane, ethane, carbon monoxide, carbonyl sulphide, and nitrous oxide. An elevated concentration of acetone in exhaled breath beyond the normal range (300–900 ppb) can serve as a diagnostic marker for diabetes, the presence of hydrogen sulphide (H_2_S) with a value exceeding 1 ppm is indicative of halitosis, and nitric oxide (NO) serves as a diagnostic standard for chronic bronchitis (threshold: >30 ppb). The normal range of ammonia (NH_3_) in the breath is from 425 ppb to 1800 ppb. An increase in the concentration of ammonia in the human breath indicates the likelihood of chronic kidney disease [233].

Various factors can influence the ratio of VOC concentration in exhaled air, mainly internal conditions and lifestyle choices (e.g., alcohol consumption, diet). VOCs are generated during catabolic processes in the human body. They are transported from tissues into the bloodstream, where they are mixed together with biochemical compounds and metabolites from different tissues. These VOCs are carried by the bloodstream to the lungs. Consequently, these gases are exhaled. Exhaled volatile organic compounds have direct associations with the body’s metabolism. People release several hundred VOCs into the air through exhalation and skin emissions [234].

To achieve early diagnosis and monitor high-risk populations, the detection of VOCs in exhaled human breath is employed. Accurate measurement of VOCs is carried out using precise methods such as spectrophotometry, gas chromatography, and high-performance liquid chromatography. However, despite their accuracy and precision, these techniques often face drawbacks, including high costs, lack of portability, and high energy consumption [235]. Other known VOC detection methods are based on optical, chemiresistive, and electrochemical principles. Gas sensors operating on chemiresistive principles are the most frequently used sensors for gas detection compared to other sensors due to their good physical and chemical properties, such as the reaction of metal oxide materials with dioxygen [236]. Semiconductor metal oxide gas sensors, also known as MOS gas sensors, measure the changes in the density of conduction electrons on the surface of the metal oxide, which is induced by chemical interactions of the semiconductor surface of the sensor and the target gas (NH_3_, alcohol, acetone, etc.). Their main advantages include their ease of use, portability, short reaction, and recovery times. Their small size and affordability must also be mentioned. They can detect a wide range of gases [237]. Traditional MOS sensors operate at high temperatures, making it challenging to apply them to wearables. Therefore, it is crucial that MOS-based sensors can operate at room temperature (RT) because it leads to extremely low power consumption. Enhancing the performance of gas sensors based on RT MOS is advantageous through the combination of MOS with conductive polymers or carbon-based materials [238].

Analysis of the composition of respiratory gases can also be performed colorimetrically, which is an analytical optical method. Colorimetric sensors are cost-effective, highly sensitive, and specific [239]. For example, previous studies have focused on breath acetone, which is a biomarker of lipid oxidation and occurs in ketoacidosis or can measure fat burning during exercise. The colorimetric sensor measures the specific reaction between acetone and hydroxylamine sulphate and has shown high accuracy and agreement with the reference device in monitoring ketosis [240]. In another article, the authors dealt with the detection of lung cancer. They found that the colorimetric sensor can identify biosignals in the breath of lung cancer patients with moderate accuracy but can be further optimized by evaluating specific histology and incorporating clinical risk factors [241,242]. Colorimetric monitoring of exhaled breath is also able to record the balance of the human organism by indicating the biomarker Nitric Oxide (NO) and thus indicate oxidative stress. NO is detected using m-cresol purple, bromophenol blue, and Alizaringelb dye and analyzed by ultraviolet-visible (UV-Vis) spectroscopy. The dye m-Cresol Purple proved capable of selectively, sensitively, and quickly sensing NO in exhaled air and thus detecting oxidative stress in the body [243]. Not only NO but also CO_2_ can be captured colorimetrically. The study by Shadid et al. [244] points to changes in CO_2_ concentrations in unhealthy people. They constructed a portable device with a smartphone-supported unit. Many approaches are only in the experimental phase, but the colorimetric approach has many advantages, which show that it is also suitable in practice.

Wearable respiratory sensors for NH_3_ detection are mainly sensors with a single signal, such as resistive, optical, or current NH_3_ sensors. Resistive NH_3_ sensors are the most frequently used sensors for breath analysis due to their simple structure, high sensitivity, and ease of manufacture. However, these sensors can be easily interfered with by environmental factors such as humidity or temperature, which can cause inaccurate results. In the study by Chen et al. [30], a dual-signal wearable sensor mask was fabricated consisting of two NH_3_ sensors (visual and resistive NH_3_ sensor) prepared by electrospinning technology. The resistive sensor of the dual-signal wearable sensor mask consists of conductive polymers, especially polyaniline. Metal oxides (such as ZnO and SnO_2_) are also widely used in resistive NH_3_ sensors. The visual NH_3_ sensor is made of bromocresol green. The advantages of the dual-signal wearable sensor are high sensitivity, fast response, and good environmental stability.

Aqueveque et al. [245] focused on improving workplace health and safety by creating an electronic respirator that captures real-time data from an integrated pressure, temperature, and relative humidity sensor and wirelessly sends it to an external platform where it can be further evaluated. Many workers working in hazardous environments wear protective respirators, and this approach could help better protect the respiratory tract and prevent disease.

### 5.2. Change in Breathing Gas Temperature, Humidity, and Pressure

Various changes in breathing gases during inhalation and exhalation enable the detection of other interesting parameters related to breathing [246,247]. Airflow detection relies on the fact that exhaled air is warmer, has higher humidity, and contains more CO_2_ compared to inhaled air. These variations can be utilized to indicate RR.

One method for measuring airflow is the use of a nasal or oronasal thermistor, which detects temperature changes between inhaled and exhaled air. This provides a semi-quantitative estimate of airflow, but its effectiveness is limited due to a high incidence of thermistor displacement [248]. Sensors measuring respiration by exhaled and inhaled air temperature use a non-invasive methodology and require a sensor, for example, between the nose and the mouth, as shown by Hurtado et al. [249], or a camera system [250]. The mean RR difference between the respiration monitor and counting was 0.4 breaths per minute (BPM). Other authors also pointed out the low cost of such a solution. They have developed a breathing sensor that is portable, easy to use, and economical. When breathing changes, a buzzer sounds to warn that something is happening to the patient, for example, during sleep [251]. Instead of a sensor near the nose, it is also possible to use thermal imaging cameras, which can record temperature changes during respiration without contact. Such solutions were also very suitable during the COVID-19 pandemic. The system can only recognize the face of the measured subject and estimate the RR. The absolute error was recorded at 0.66 bpm [252]. Hsu and Chow [253] introduced an RR monitoring system utilizing a thermal sensor designed to monitor infants. It is noteworthy that this system includes a mask, enabling it to operate without direct contact with the child’s skin. The sensor identified temperature variations induced by respiration, and the collected data underwent real-time correction and analysis using a personal computer connected to the central nursery. Yu et al. [254] described passive wearable sensors for monitoring RR from heat. They used micro thermoelectric generators (μTEGs) capable of monitoring and detecting diseases related to breathing. The μTEG design is unique and provides fast response and real-time measurement. Zhao et al. [255] proposed an all-fiber microcantilever-based breath sensor that was fabricated at the fiber tip by femtosecond laser-based two-photon polymerization microfabrication. A micro Fabry–Pérot (FP) interferometer was formed between the microcantilever and the end side of the fiber. The sensor has excellent thermal stability and has been mounted on a surgical mask where it has demonstrated the ability to detect different breathing patterns and, thus, is suitable for RR monitoring. The method is also suitable for application in an MRI environment.

Resistive humidity sensors operate by detecting changes in electrical conductance or resistance in response to variations in the surrounding water concentration. Recently, humidity sensors have been found to be applied in the field of respiration monitoring. Wang, et al. [256] demonstrated amicron line humidity sensor based on PEDOT:PSS for respiration monitoring. The PEDOT:PSS (poly (3, 4-ethylenedioxythiophene):(polystyrene sulfonate)) sensor prepared by the femtosecond laser printing method was small, which could lead to easy integration with different wearable devices. The sensing system featured good stability, low humidity hysteresis, and fast response–recovery time. Zhou et al. [257] also demonstrated real-time monitoring of human respiration using a humidity sensor based on PEDOT:PSS in the format of nanowires on the PET substrate. Their experiments also demonstrated high sensitivity, an ultrafast response, and excellent mechanical durability and robustness. Güder et al. [258] developed a system for RR measurement based on humidity sensors printed on paper. The paper sensors were fabricated by digitally printing graphite ink using a ball-point pen and craft cutter/printer onto paper. The sensor was a simple two-electrode electrochemical cell, in which water was electrolyzed by applying an electrical potential between the electrodes. They placed a pure cellulose paper-based humidity sensor in textile masks. The optimized textile mask effectively monitored the respiratory activity of the subject. The integration of the humidity sensor into the textile mask was also used in another study [259], where, instead of graphite ink, PEDOT:PSS ink was used. PEDOT:PSS, with a conductive ink specifically formulated in the study, was printed on polyamide-based taffeta label fabric by an inkjet printing method. The humidity sensor distinguished between fast/deep and nose/mouth breathing. The flexible and wearable humidity sensors prepared based on conductive polymer PEDOT:PSS ink or graphite ink may also be good candidates for future respiration-monitoring health applications, above all for their flexibility and low-cost production using printing technologies. Other wearable humidity sensors utilizing a porous graphene network with the capability to detect moisture have undergone testing for respiration analysis [260]. These sensors are attached to the body in the form of a mask and exhibit the ability to sense various aspects of human breathing, including detecting apnea, analyzing speech patterns, and identifying wheeze rhythms. Honda et al. [32] presented a highly stable humidity sensor for home sleep apnea monitoring. This system, in the form of a mask for home use, is wireless and designed for long-term monitoring.

Another sensor used for measuring the respiratory rate is the nasal pressure transducer. Nasal pressure offers a more accurate measure of airflow as it is based on the actual volume of air exhaled [261,262]. This measurement can be obtained through a nasal cannula, mouthpieces, or facemasks. However, some patients may find the sensor uncomfortable, and the collector can potentially impact respiratory activity by increasing dead space [263]. The facemask introduced in [264] is designed to measure respiratory impedance and is intended for both home and clinical applications. This innovative solution incorporates two pressure transducers, two low-power consumption fans, a field-programmable gate array, and a real-time processing engine. The device utilizes the forced oscillation technique (FOT), a non-standardized lung function test. This technique involves using fans to introduce a periodic sinusoidal air pressure signal and measuring the opposing force produced by the respiratory tract. Manoni et al. [265] designed a wireless system for home monitoring and tracking of specific breathing disorders during sleep, which are manifested by episodes of apnea and hypopnea of central or obstructive origin. They explored several positions, but the best place to place the device was the nose so that the pressure sensor could detect the RR.

### 5.3. Wearable Spirometry

Pulmonary function testing (PFT) constitutes non-invasive tests used for the diagnosis of various pulmonary disorders that affect a large amount of people worldwide. The first part of pulmonary function testing is spirometry [266]. Spirometry is an objective and non-invasive diagnostic test that is sensitive to early changes, and it is useful for disease progression. This test is easy to perform. The quality of spirometry measurements is very important. Therefore, it is necessary to perform a spirometry test as aptly as possible; otherwise, the results could be misinterpreted. A spirometer serves to measure the volume, flow, and duration of ventilated air, and then these parameters are processed into output curves. Spirometry parameters provide important information about lung function [267]. It is helpful in determining obstructive airway disorders (asthma and chronic obstructive pulmonary disease) but it is less useful in evaluating restrictive diseases [266]. The physical parameters obtained from spirometry measurements include forced expiratory volume in 1 s (FEV_1_), forced vital capacity (FVC), the FEV_1_/FVC ratio, and others (vital capacity, forced expiratory flow 25–75%, peak expiratory flow, and inspiratory vital capacity) [268]. FVC is the maximum volume of air that can be exhaled. FEV_1_ is the maximum amount of air exhaled forcibly during the first second [269]. Spirometry is usually operated in a medical environment such as a lab or clinic. However, such a medical environment cannot reflect the participant’s ordinary daily life. Therefore, it is essential to focus on spirometry sensors integrated into wearable devices. The minimalization of wearables will ensure the implementation of these measurements anywhere and at any time.

Reference [270] represents a turbine-based MEMS sensor suitable for integration into wearable devices for spirometry measurements. The dimensions of the sensor are 20 × 20 × 2.5 mm and it consists of a turbine, built-in multipole permanent ring magnets, and two stators with one-phase copper micro coils. Tangential air flow causes the rotation of the turbine, which leads to voltage generation due to electromagnetic induction. This mechanism of sensing the breath flow represents benefits such as insensitivity to ambient temperature, humidity, and gas content. The sensor can easily be placed in a wearable device due to these design features. The lung volume and flow rate are the parameters that can be obtained. Another alternative to measuring spirometry parameters by wearable sensors is the system described in [271]. The system contains a BME280 sensor, which can measure barometric pressure, relative humidity, and temperature while breathing. Due to its compact size, the sensor can be integrated into any wearables, whether in the form of open-air headsets or integrated into respiratory/training masks. It is possible to estimate breath volume using this wearable spirometry system. Zhou et al. [272] presented a face mask with a differential pair of barometric sensors, which monitor the participant’s breath. This setup was used for higher accuracy in the measurement. Therefore, the mask includes two barometers located inside and outside the mask. This work points to the utilization of barometric pressure sensors as a cheap version of breath sensing with compact dimensions, which provides the possibility of applications in wearable facemasks for continuous breathing volume monitoring.

A cardio-respiratory monitoring system CoRSA is presented in [273]. The CoRSA system is based on a pressure sensor implemented inside the respiratory mask and a pulse-oximeter located on the earlobe. The CoRSA device measures HR, SpO_2_, RR, and RV. Metamax 3B Cortex Medical is a commercial mobile spiroergometry system specifically crafted for professional outdoor use, capable of functioning under various sports conditions. The base of the system consists of an oronasal sensing training mask. Its benefit is its low weight and convenient system package. The device boasts an impressive battery life of approximately 6 h, ensuring extended usability during activities. This system is designed to provide a comprehensive range of measurements and calculations. It encompasses the assessment of various parameters, including heart rate, blood pressure, ECG, gas exchange parameters, spirometry parameters, and ventilation parameters [274].

### 5.4. Composition of Blood

Blood gases, including oxygen (O_2_) and carbon dioxide (CO_2_), are vital indicators of the body’s acid–base balance and respiratory function. Oxygen is essential for cellular metabolism, while carbon dioxide is a byproduct of metabolism that needs to be efficiently eliminated. The balance between oxygen and carbon dioxide is crucial for maintaining homeostasis in the body.

Blood oxygen saturation (SpO_2_) measures how much hemoglobin is bound to oxygen compared to how much hemoglobin remains unbound [275]. Hemoglobin can carry up to four oxygen molecules. SpO_2_ is a basic parameter that is considered by default in patient care. Oxygen is very important for the human body due to its necessity in metabolic cellular processes, and a drop below vital levels can have an acute adverse effect on organ systems. Decreased saturation may indicate lung diseases. The measurement of saturation is based on the PPG method, in which the reflection or transmission of light changing with the volume of blood in the microvascular area of the tissue is recorded. Oxygenated hemoglobin absorbs more infrared light. This approach has become increasingly popular for various wearable devices that can be used in both hospital and home environments [176,177,276,277]. They consist of a light source and a photodetector. The commonly recorded PPG signal by wearable devices is prone to artifacts, so Beh et al. [177] described in their publication a machine-aided signal quality assessment (SQA) system. This method would increase the accuracy of monitoring PPG signals. A deep-learning approach to SpO_2_ estimation from PPG signals is coming to the fore. A smart device with a PPG sensor can come in various forms, such as a classic pulse-oximeter, a patch that attaches to the chest [278], a ring [279,280,281], an in-ear wearable sensor [282], a device attached to the bottom of the foot [283], or a portable wearable watch [284,285].

Carbon dioxide (CO_2_) plays a crucial role in blood pH regulation and influences hemoglobin’s affinity for oxygen. Monitoring the partial pressure of CO_2_ (PCO_2_) is significant in the medical diagnosis and treatment of respiratory and metabolic diseases. Common methods for assessing carbon dioxide partial pressure (PaCO_2_) include invasive arterial blood gas (ABG), arterialized capillary blood gas (CBG), and peripheral venous blood gas (VBG). Deviation from the norm in carbon dioxide pressure can disturb the acid–base balance of the body, leading to hypocapnia or hypercapnia resulting in various disorders (respiratory, metabolic, or neurological). The drawbacks of these methods include the pain associated with the procedure, the requirement for health professionals, impaired skin integrity, and the unsuitability of these blood gas analysis methods for continuous monitoring [286].

Non-invasive alternatives for assessing PCO_2_ include capnography and transcutaneous CO_2_ monitoring. Capnography refers to the sensing of the end-tidal partial pressure of carbon dioxide (PetCO_2_) in the expired gas. Transcutaneous monitoring serves the continuous monitoring of oxygen (TcO_2_) and carbon dioxide (TcCO_2_), which diffuses through the skin [287]. Various highly vascularized skin locations are utilized for the placement of CO_2_ sensors for transcutaneous CO_2_ monitoring, e.g., the earlobe. This method can be employed to estimate the arterial partial pressure of oxygen and carbon dioxide. The diffusion of blood gases is typically low. Hence, achieving accurate sensing requires heating the skin to 42 °C or higher to enhance sufficient CO_2_ diffusion [31]. Traditional transcutaneous CO_2_ sensing uses electrochemical sensors that monitor changes in CO_2_ penetrating the skin. However, these traditional sensors consist of bulky electronics and require frequent calibration, making them unsuitable for monitoring outside clinical environments [288]. Another technique for transcutaneous CO_2_ monitoring utilizes non-dispersive infrared (NDIR) gas sensors. NDIR technology is based on the Beer–Lambert law, which is employed to determine the concentration of chemical substances with light absorption capacity. Carbon dioxide absorbs infrared light at a wavelength of approximately 4.26 µm, resulting in the attenuation of infrared radiation passing through a gas sample containing CO_2_ [289]. In a previous study [288], the authors introduced a compact prototype with the potential for use as a wearable transcutaneous CO_2_ device in healthcare applications. This prototype utilizes IR LED and thermopile reading circuits. The thermopile within the sensing unit transforms IR intensity information into a voltage value, utilizing temperature-sensing thermocouples. The in vitro measurement results show the successful monitoring of PCO_2_ within the 0–120 mmHg range, encompassing typical human values of 35–45 mmHg. Tipparaju et al. [286] developed a wearable wristband device based on a miniaturized NDIR sensor for continuous transcutaneous CO_2_ monitoring. This miniaturized NDIR sensor is distinguished by its exceptional accuracy, long lifespan, and low power consumption. To further enhance its performance, the authors developed a hydrophobic membrane with high CO_2_ permeability, effectively mitigating humidity interference. This innovation ensures the reliable and continuous transcutaneous blood CO_2_ tracking capability of the wristband without the need for skin heating. The device consists of a plastic body, where the NDIR sensor is placed, and a gas chamber with a volume of 1 ml allowing the accumulation of diffusing CO_2_ through the skin. An O-ring, positioned atop the gas chamber, serves as both a cushion between the wristband and the skin and ensures an airtight seal. This design prevents any gas leakage, maintaining the integrity of the system and preventing the entry or escape of gases into or from the ambient air.

Another alternative to transcutaneous blood gas sensing is described in another study [290]. This innovative miniaturized prototype for transcutaneous carbon dioxide monitoring applies the principle of fluorescence. The chemical reaction occurring in the thin fluorescent film in response to CO_2_ is converted into an optical signal. This conversion is achieved by optically stimulating the thin film with a light-emitting diode (LED), followed by the measurement of fluorescence intensity to quantify PCO_2_. Transcutaneous CO_2_ sensors utilizing luminescent materials present numerous advantages, including precise CO_2_ level detection and considerable potential for miniaturization. The authors [31] designed a compact and lightweight prototype to measure the partial pressure of CO_2_. The sensing mechanism involves stimulating and detecting the fluorescent response of the CO_2_-sensing film. Shahed et al. [291] presented another study that focused on the estimation of capnography from a PPG signal, employing a sequence-to-sequence prediction perspective. In this design, the encoder module plays a crucial role in converting the input PPG signal into a more refined representation within a reduced dimensional space. A notable innovation in this study involves the incorporation of a recurrent block, consisting of long short-term memory units positioned between the encoder and decoder. The final component is the decoder, where the desired output signal is reconstructed into a capnograph signal.

**Table 4:** Parameters of exhaled and blood gases related to respiration.

Sensor Type	Application	Sensing Element	Key Parameters	Ref.
Face mask	Detection of VOCs ^1^	Chemiresistive MOS ^2^ sensor	Excellent stability, High response value, Low cost	[238]
Face mask	NH_3_ detection	Optical resistive sensor	High sensitivity, Fast response, Good environmental stability	[30]
Spirometry face mask	HR, Blood pressure, ECG, gas exchange, spirometry	Turbine-based NDIR CO_2_, fuel-cell-type O_2_ sensor, pressure sensor	Commercial mobile spiroergometry, Low weight, 6-h working time	[274]
Spirometry face mask	RR ^3^ and RV ^4^	Turbine-based MEMS sensor	Insensitivity to ambient temperature, humidity, and gas content	[270]
Open-air headset for spirometry	RR and RV	Pressure, humidity, and temperature sensor	Compact size, 96% accuracy for face mask, 82% accuracy in open-air headset	[271]
Spirometry face-worn garments	RR, RV, FVC ^5^, IRV ^6^, ERV ^7^, IC ^8^	Differential pressure sensor	Cheap version of sensing, Error margins for FVC 2–3% and for RV 1–3%	[272]
Spirometry mask with and earlobe type PPG	RR, RV, HR and SpO_2_, activity	Pressure sensor	Pressure, humidity, and temperature sensor BME280, IMU ^9^ for activity tracking	[273]
Face mask	RR, sleep apnea	Humidity sensor	Bluetooth connection	[32]
Face reusable respirators	RR, fit of the filter estimation, Contamination lvl	Pressure, temperature, relative humidity sensor	Protect workers from harmful dust, smoke, gases, and vapors	[245]
Nose sensing	RR, apnea and hypopnea	Pressure sensor	PPG, ACC, Microcontroller, Bluetooth	[265]
Sensor under the nostril and near the mouth	RR	Micro thermoelectric generators	Ultra-thin vertical structure-rapid heat conduction, Horizontal high-density integration-transient response and high fill speed, 28-pair microthermoelectric legs	[254]
Surgical mask	RR	Optical fiber	Thermally stable, Compact, Flexible, MRI conditions	[255]
Patch-like device	SpO_2_	PPG	Emergency situations, Real-time monitoring	[277]
Ring	SpO_2_, HR, HRV	PPG	MAX30102, Error rates lower than 2.5%	[278]
Ear Monitor	RR, SpO_2_, HR, temperature	PPG	Bluetooth, MAX30100, TMP006 infrared sensor, analyzing respiratory sinus arrhythmia (RSA)	[281]
Watch	SpO_2_, HR	PPG	Bluetooth 4.0	[284]
Transcutaneous sensing	Partial pressure CO_2_ monitoring	NDIR ^10^ sensor	Range 0–120 mmHg, Thermopile reading circuits	[288]
Transcutaneous sensing in wristband	Partial pressure CO_2_ monitoring	NDIR sensor	No need for skin heating, High accuracy, Long lifespan, Low-power consumption	[286]
Transcutaneous monitoring	PtcCO_2_ monitoring	Optical fluorescence thin film sensor	Range 0–75 mmHg	[290]
Transcutaneous sensor on a forearm	PtcCO_2_ monitoring	Optical fluorescence sensor	Highly sensitive in the CO_2_ range (0–50 mmHg), Insensitive to humidity	[31]
PPG sensor	Capnography measurement	Capno-base dataset	Deep neural network, Low cost, MSE ^11^ 0.21, Cross-Correlation 0.946	[291]

^1^ Volatile organic compound, ^2^ Metal oxide semiconductor, ^3^ Respiratory rate, ^4^ Respiratory volume, ^5^ Forced vital capacity, ^6^ Inspiratory reserve volume, ^7^ Expiratory reserve volume, ^8^ Inspiratory capacity, ^9^ Inertial measurement unit, ^10^ Non-dispersive infrared, ^11^ Mean square error.

Devices measuring parameters of exhaled and blood gases have a wider scope and can detect various gases and respiratory parameters. They provide valuable information about exhaled gases. They are often in the form of breathing masks (CO_2_ and NH_3_) or spirometers or evaluate blood parameters (SpO_2_ through the skin). Breathing masks have the advantage of being located close to the nose and mouth, providing a comprehensive view of respiratory gases (CO_2_ and NH_3_), humidity, and temperature, which creates a picture of the overall health of the respiratory tract. However, respirators are quite uncomfortable to wear for long periods of time, and improved designs and feedback can solve this problem and help optimize wearing discomfort.

When evaluating blood parameters, it is great that sensors can non-invasively monitor blood gases continuously without blood sampling. They are usually in the form of smartwatches or fitness trackers. The disadvantage is usually the low diffusion of gases (mainly CO_2_) on the skin surface as well as a more limited measurement range compared to other more complex sensors. The low diffusion of gases is accelerated by a local increase in temperature. However, this increases consumption and demands for precise regulation and also increases discomfort. The limited lifetime of electrochemical sensors, their more frequent calibration, and requirements for more complicated electronics are also disadvantageous.

Spirometers are suitable for the comprehensive monitoring of breathing, but they also offer a detailed assessment of lung capacity, volume, and air flow that other sensors cannot measure. Determining these parameters helps in the management and diagnosis of respiratory diseases. The use of spirometers requires training and skills, which can be a disadvantage of this approach to measuring respiration, as well as the inability to continuously measure changes in respiration.

## 6. Brief Summary

In the article, we described many methods for measuring respiration, either with wearable or remote electronics. Table 5 shows a simplified comparison of these different sensors and respiration detection methods. The parameters are averaged and may vary slightly in specific applications.

In summary, we can conclude that the choice of a suitable sensor is subject to a compromise between the accuracy of the measured data and the comfort of the wearer, while efforts are being made to improve both sides. The chosen method for measuring respiration depends on the application of the device. For example, chest straps excel in accuracy and stability, so they are ideal for continuous monitoring. They are preferred for use in sports and fitness, where stability and precision are crucial even in movement. In contrast, other devices that are capable of multisensory measurement are more suitable for general health monitoring.

The integration of SCG and BCG sensors into devices offers wearers the possibility of all-day continuous monitoring. They are innovative approaches that offer the capture of subtle movements, but the disadvantage is the complexity of the interpretation and their susceptibility to interference, which can be solved with more sophisticated signal-processing algorithms. The future will hopefully bring an increase in the resistance of sensors to external interference, optimization of their integration into various forms, and overall effort for a better user experience. The choice of these methods also depends on the specific use, whereby the emphasis is always placed on comfort, versatility, and obtaining high-quality, informative data. Impedance-based sensors are a significant alternative for continuous monitoring. The advantages are the lower sampling frequency and lower energy consumption. In contrast, disadvantages include problems with sensitivity and accuracy, which offer future opportunities to work on improvements in optimization and algorithms for signal processing and minimizing interference by movement or external factors. In this way, it will be possible to ensure accurate and reliable data.

Optical sensors are suitable for wearable and remote devices due to their accuracy, high sensitivity, and non-interference in an electromagnetic environment. Their weakness is fragility, more complex signal processing, and, therefore, higher costs. Equally, in this case, the development of perfect algorithms to simplify data interpretation and efforts to increase resistance will help these sensors look even more attractive to users. In the future, it is possible we can expect improvements in energy consumption, sampling frequencies in the field of algorithm development, and, in general, putting optical sensors in a better place in the field of respiration monitoring.

What is interesting about radar sensors is their non-contact and versatile use, while the challenge for the future is the improvement of signal-processing algorithms, a reduction in signal interference, and integration into various smart devices in the home or applications for assisted living.

Camera systems allow non-contact breathing monitoring with wide applicability and cost-effectiveness. In addition, thermal cameras are effective for measuring respiration even in low light and are less personal. Their accuracy and measurement capabilities can be increased by using modern technologies such as artificial intelligence and advances in image processing. Algorithm optimization will also help improve the obtained data and give a more accurate result or obtain more respiratory data from the recording. In the future, development may also move in the direction of solving privacy protection and improving the affordability of this respiration measurement method.

Widely available ECG and PPG sensors in devices are also a good and practical solution for measuring respiration. Their advantage lies in their wide availability, the possibility of continuous monitoring, and non-intrusive characteristics. They are therefore suitable for a wide range of users. Their further development is based on improvements in the field of signal processing, ensuring accuracy, and efficiency and the use of artificial intelligence to improve the ability to recognize and interpret different breathing patterns.

Acoustic sensors are also very popular with users. They constitute a versatile solution to recording respiration. Another advantage is the real-time recording of breathing sounds and, thus, the possibility of monitoring various respiratory diseases. They are, therefore, very important tools in various healthcare and wellness applications. In the future, to increase the accuracy of respiratory data, the problems of signal interference by the surrounding environment will be solved, and algorithms and techniques for processing complex signals will be improved. With acoustic sensors, it is very important to filter out ambient noise unrelated to breathing.

Sensing parameters from exhaled and blood gases is possible with devices such as breathing masks, which have the advantage of obtaining respiratory parameters that other sensory devices cannot, such as the levels of specific respiratory gases. In the future, in this direction, the analysis of measured data, more complex and personalized breathing monitoring solutions, the comfort of wearing masks, the range of measured parameters, and the user-friendliness of spirometers can be improved. These devices are important to support the early detection of respiratory health problems.

## 7. Conclusions

In this article, we have conducted an extensive review of respiration sensors in use or prospective for wearable and remote electronics. We identified four main methods: measuring movements related to respiratory effort, deriving respiration from the parameters of the cardiovascular system, listening to acoustic manifestations of breathing, and sensing the parameters of exhaled and blood gases that are related to respiration. We clarified the basic physiological principles and added the basic advantages and disadvantages of individual methods.

The main limitation of the article is that we could only focus on a certain part of wearable and remote technology. The selected devices describe only a low percentage of the total research. For example, we largely ignored material research. If we analyzed the detailed use of organic materials or optical fiber technology, the article would have given rise to two more sequels. We also did not go into full detail about the technical aspects of the research. Because of the size of the article, we could only focus on the most promising types. If the reader is seeking more, further research on many more sensors can be found in the overview sections of most of our mentioned articles. We also draw attention to the review by Hussain et al. [247], which covers a wide range of wearable respiratory sensors and specifically focuses on the materials used.

As can be seen from the analysis of the market and literature, on one side, there is a huge interest in research, but on the other side, the quality and number of wearable and remote devices in the current market are somewhat weaker. This places great demand on the translation of promising research into practice. We believe that this study will be helpful and will serve as a springboard not only for respiratory and telemedicine research but also for the introduction of other promising devices into practice.

## Figures and Tables

**Table 5:** Comparison of properties of different wearable and remote respiration-sensing technologies.

Method	Advantages	Disadvantages	Measured Parameters	Accuracy	Application	Sampling Frequency	Convenience
Chest belt	High accuracy cost-effective, long battery life	Uncomfortable for long-term use, may not capture subtle movements	RR, RV, apnea	Generally high for basic monitoring	Well-suited for sports and fitness applications, sleep monitoring	<100 Hz	Moderate
Patch	Comfortable, small size, suitable for long term monitoring	Precise placement and close contact with skin is crucial	RR, RV	Generally high for basic monitoring	Well-suited for sports and fitness applications, disease progress	<100 Hz	High
SCG/ BCG	Capture subtle movements, patterns	Complexity in interpretation, susceptible to interference	RR, RV, respiratory patterns	Variable, may require validation for clinical use	Cardio-respiratory dynamics, Sleep monitoring	0.1–5 kHz	Moderate
Impedance	Nonintrusive, low power consumption	Susceptible to interference, proper contact is crucial	RR, RV	Variable, affected by skin contact	Suitable for continuous monitoring	0.03–32 kHz	Moderate
Optical fiber	Capture subtle movements, comfortable, resistant to EMG	Sophisticated signal processing, relatively expensive, fragile	RR, RV	Generally good for basic monitoring	Integrated into clothing or beds, monitoring in MRI or CT	0.01–3 kHz	High
Camera	Non-contact, captures multiple parameters	Privacy concerns, limited accuracy in certain conditions	RR, RV, temperature	Moderate, affected by lighting and resolution	Continuous monitoring in controlled environments	30–100 Hz	Remote
Radar	Non-contact, captures motion through clothing	Limited accuracy in certain situations	RR, RV	Moderate, affected by environment	Continuous monitoring in controlled environments	Operational frequency 1.8–24 GHz	Remote
EDR	Continuous monitoring, additional cardio data	Indirect measurement, accuracy influenced by artifacts	RR, RV	Generally good for trends monitoring	Combined cardio and respiratory assessment	50–500 Hz	High
Acoustic	Non-invasive, cost-effective	Ambient noise interference, may not be suitable for all settings	Respiratory sounds, RR, airflow	Good for certain applications (e.g., diagnosing respiratory conditions)	Cough, asthma, apnea detection, remote patient monitoring, smartphone apps	0.01–22 kHz	Moderate
Gases	Comprehensive view of respiratory parameters	Limited scope, uncomfortable	SpO_2_, CO_2_, NH_3_, humidity, temperature	High for clinical settings	Diagnosing specific respiratory and metabolic conditions	-	Low

## Data Availability

Data sharing is not applicable.
